# Biomimetic Cell Membrane‐Coated MOFs System for Targeted Cancer Therapy

**DOI:** 10.1002/advs.202521580

**Published:** 2026-03-12

**Authors:** Qilu Wu, Jia Song Deon Chon, Yuxiao Feng, Kang Liang, Shiming Li, Can Yang Zhang, Jun Ge

**Affiliations:** ^1^ Key Lab for Industrial Biocatalysis, Ministry of Education Department of Chemical Engineering Tsinghua University Beijing China; ^2^ School of Chemical Engineering The University of New South Wales Sydney NSW Australia; ^3^ Graduate School of Biomedical Engineering The University of New South Wales Sydney NSW Australia; ^4^ Beijing Tongren Eye Center Beijing Ophthalmology & Visual Sciences Key Laboratory Beijing Tongren Hospital Capital Medical University Beijing China; ^5^ Institute of Biopharmaceutical and Health Engineering Shenzhen International Graduate School Tsinghua University Shenzhen China; ^6^ State Key Laboratory of Green Biomanufacturing Beijing China; ^7^ Center For Synthetic and Systems Biology Tsinghua University Beijing China

**Keywords:** biomimic, cancer therapy, cell membranes, drug delivery, metal‐organic frameworks

## Abstract

The integration of metal‐organic frameworks (MOFs) with cell membrane coatings has emerged as a revolutionary strategy to enhance the therapeutic efficacy of cancer nanomedicine. In this review, we systematically summarized recent advances in cell membrane‐camouflaged MOF as drug delivery systems, focusing on their unique advantages in tumor targeting, immune evasion, and biocompatibility. By emulating the characteristics of natural cells, these biomimetic systems demonstrate enhanced circulation time, increased accumulation within tumors, and decreased uptake by macrophages. A critical analysis was conducted on a variety of membrane sources, including cancer cells, red blood cells, and macrophages. The analysis focused on the functionalities of these sources in tumor homing and immune modulation. The following design strategies are of particular interest: hybrid membrane engineering and stimuli‐responsive drug release mechanisms. This field demonstrates considerable potential for combination therapies integrating chemotherapy, immunotherapy, and photothermal modalities. In the future, the development of new drugs should prioritize intelligent drug release systems and personalized treatment through advanced biomaterial engineering.

## Graphical abstract

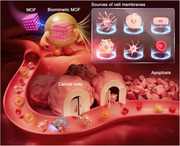

## 1 Introduction

Cancer is a highly complex and heterogeneous disease, primarily characterized by abnormal cell proliferation, uncontrolled growth, and potential metastasis to distant sites. According to data from the World Health Organization (WHO), approximately 20 million people are diagnosed with cancer globally each year, and about 10 million die from the disease, making cancer the second leading cause of death worldwide, following cardiovascular diseases [1]. Common types of cancer include lung cancer, breast cancer, colorectal cancer, and prostate cancer, with lung cancer being the leading cause of cancer‐related deaths globally, accounting for 23% of all cancer fatalities [2]. This high incidence and mortality rate places a significant burden on global healthcare systems, underscoring the urgent need for effective and safe treatments. Traditional cancer treatments such as chemotherapy, surgery, and radiotherapy remain the primary approaches in current practice [3, 4, 5]. Although chemotherapy and radiotherapy are both highly effective in killing cancer cells, they are also associated with severe side effects that can have a profound impact on the patient's recovery [6, 7, 8]. Moreover, surgery is limited to localized tumors and is often ineffective for metastatic cancers [9]. These conventional treatments often result in significant suffering for patients and may not consistently achieve the desired therapeutic outcomes. Recently, novel therapies based on molecular and metabolic mechanisms, such as RNA interference (RNAi), phototherapies, immunotherapy and chemodynamic therapy, have provided more precise approaches to cancer treatment [10, 11]. However, drug delivery still faces challenges such as biological barriers, low targeting efficiency, drug degradation and multidrug resistance, which have a negative impact on the efficiency and effectiveness of treatment [12, 13, 14, 15]. Therefore, designing a highly stable and targeted delivery system is a crucial area of research in biomedicine.

Although several nanomedicines have achieved clinical translation such as Doxil and Abraxane demonstrated breakthrough clinical success, their overall efficacy in solid tumors remains constrained by certain limitations [16]. First, conventional nanomedicines rely heavily on the enhanced permeability and retention (EPR) effect to achieve tumor accumulation, yet the highly heterogeneous human tumor microenvironment renders this passive targeting approach ineffective [17]. Additionally, the immunogenicity of polyethylene glycol (PEG) as a stealth coating has emerged as a critical clinical concern. Studies indicate that PEG induces the production of anti‐PEG antibodies and accelerates the phenomenon of accelerated blood clearance, leading to rapid drug elimination and potentially triggering hypersensitivity reactions [18]. Furthermore, traditional nanomedicines often struggle to penetrate complex biological barriers, most notably the blood‐brain barrier (BBB). This barrier consistently blocks over 98% of neurotherapeutic drugs, making aggressive malignancies like glioblastoma highly challenging to treat effectively [19]. These persistent limitations have driven the development of next‐generation bionic platforms, such as cell membrane‐coated metal‐organic frameworks (MOFs), which leverage endogenous cellular characteristics to achieve superior immune evasion, homotopic targeting, and barrier penetration.

The emergence of metal‐organic frameworks (MOFs) provides new opportunities for drug delivery in disease treatment. MOFs are porous materials formed by metal ions or clusters coordinated with organic ligands [20]. The studies of MOFs began in the 1990s and have rapidly developed in recent decades. American chemist Omar Yaghi first proposed the porous structures composed of metal ions and organic ligands, laying the foundation for MOFs [21]. In recent years, researchers have explored various applications of MOFs, including gas storage, catalysis, biosensing, and bioimaging [22, 23, 24, 25]. Compared to other porous materials, MOFs offer several distinct advantages: (1) MOFs have high porosity and surface area, facilitating higher loading capacities; (2) They exhibit structural tunability, allowing changes in physical and chemical properties by altering metal ions or clusters and organic ligands; (3) The controllable coordination bonds between metals and organic ligands impart biodegradability, improve the release of loaded molecules, and make MOFs suitable for drug delivery applications [26, 27, 28, 29, 30, 31]. Despite MOFs' high surface area and tunable pore structures, achieving precise drug release in vivo remains challenging, especially for targeting specific tissues such as tumors [32]. Some MOFs may be rapidly cleared by the body's mononuclear phagocyte system (e.g., liver, spleen), which can reduce drug delivery efficiency to the target site [33].

In addition, a new lipid shell‐based delivery system has recently emerged, with a shell consisting primarily of natural or engineered cell membranes [34]. By utilizing cell membranes from various sources, such as cancer cell membranes, platelet membranes, and red blood cell membranes, a novel type of “membrane‐encapsulated nanoparticle” has been developed [35]. These carriers demonstrate enhanced biocompatibility, immune evasion ability, and targeting capabilities when delivering macromolecules such as small molecular drugs, proteins and nucleic acids [36]. In another study, genetic engineering was widely utilized to enhance the functionality of cell membrane‐coated nanoparticles (CNP) [37, 38]. Zhang and his team demonstrated a modular CNP functionalization method based on genetic engineering that allows for the incorporation of multiple ligands (SpyCatcher and SpyTag) on the surface of nanoparticles to improve membrane targeting [38]. The result shows that the surface of nanoparticle is engineered to load a variety of biomolecules, allowing the nanoparticle to be customized for targeted drug delivery [38]. Therefore, membrane design technologies encompass various approaches and are highly advanced, offering significant potential for drug delivery applications through the engineering of cell membranes.

Fortunately, a biomimetic strategy was developed for MOF‐based drug delivery systems. The fabrication of cell membrane‐coated nanoparticle generally follows the standard “top‐down” approach established by Zhang et al. [39]. Specifically, cell membranes are isolated via hypotonic lysis followed by differential centrifugation to remove intracellular contents and recover purified plasma membrane vesicles. To ensure the integrity and functionality of the biomimetic coating, successful extraction and encapsulation are rigorously verified through a triad of characterization methods. Transmission Electron Microscopy (TEM) often applied to visualize the characteristic core‐shell structure (typically showing a ∼7–10 nm lipid halo) and Dynamic Light Scattering (DLS) able to monitor the characteristic size increase and zeta potential reversal, indicating the formation of a stable, right‐side‐out membrane coating. In biological characterization, SDS‐PAGE and Western Blotting confirm the retention of key membrane‐specific surface antigens (e.g., CD47, E‐cadherin) and the exclusion of intracellular impurities. Specifically, using the phospholipid bilayer structure of cell membranes as the camouflaged shell of MOF materials can effectively fool the body's clearance systems, ensuring that the circulation time of MOFs can be increased in vivo [40]. It has been found that there is a strong affinity between homotypic circulating tumor cells. This affinity leads to cell aggregation related to the recognition and interaction of proteins on the cell membrane [41]. Therefore, leveraging the unique advantages of cell membranes to coat MOFs for drug delivery presents an effective strategy to address this issue. Scientists have designed different biomimetic MOFs for drug delivery, representing a significant advancement in drug delivery systems [42]. This strategy harnesses the advantages of different membranes to achieve more effective targeted delivery, enhanced biocompatibility, and improved drug stability. It provides a safer and more effective treatment option in precision medicine, gene therapy, and tumor immunotherapy, promoting new drug development and clinical application. Compared to traditional carriers, cell membrane‐coated systems can better mimic the functions of natural biological entities, enhancing targeting ability, immune evasion, and delivery efficiency. Membranes from cancer cells, red blood cells (RBCs), platelets, macrophage cells, and stem cells show great potential, especially in tumor therapy, anti‐inflammation, and immunotherapy. Table 1 summarizes the application of biomimetic MOFs in cancer therapy. In the future, with technological advancements, these membrane carriers are expected further to promote the development of precision medicine and personalized treatment.

**TABLE 1:** The summary of biomimetic MOFs in cancer therapy.

Membrane Source	Name	Cargo	Cell line	Feature	Refs.
Cancer Cell Membrane	mPAGT	l‐Arginine & Gox	4T1 cells	O_2_ generation by nanophotosensitizers and ROS‐mediated NO release, and inducing apoptosis.	[43]
	DOX@COD‐MOF@CCM	DOX, COD	MCF‐7/ADR cells	Decrease the rigidity of drug resistant and restore the sensitivity of multidrug‐resistant cells to DOX.	[44]
	AMR‐MOF@AuPt	R848 molecules	Hep1‐6 cell	Synergistic sonic immunotherapy inhibits tumor growth and induces robust systemic long‐term immune memory.	[45]
	[Zr‐TCPP(TPP)/R837@M]	R837 molecule	4T1 cells	Achieve in vivo synergy with anti‐CTLA‐4 immune checkpoint blockade to reverse the immunosuppressive tumor microenvironment.	[46]
	CAMEL	Plk1 siRNA	Hela cells	The delivered siRNA effectively inhibited the expression of PLK1 and suppressed tumor growth in vivo.	[47]
	MCM@UN	NHWD‐870	TNBC cell	Remodel the tumor immune microenvironment and inhibit the malignant behavior of TNBC.	[48]
	C‐Z@CM	ZnO	HeLa cells	Coupled with the cascade generation of ROS and RNS, accompanied by glutathione depletion.	[49]
	Hm@TSA/As‐MOF	TSA&AS	Hepatocellular carcinoma	Upregulate the abundance and activity of tumor‐infiltrating T lymphocytes (TILs) and synergize with PD‐1 antibodies against HCC.	[50]
	mFe(SS)/DG	GOx, DOX	4T1 cells	ROS‐ferroptosis‐Glycolysis‐based regulation integrating tumor metabolism and immunity.	[51]
	cMn‐MOF@CM	CpG oligodeoxynucleotide	B16‐OVA cells	Sonodynamic therapy (SDT) combined with an anti‐PD‐1 antibody induces stronger systemic immune responses.	[52]
	PCN‐PL@CM	piperlongumine	3T3 cell, CT‐26 cell	Photodynamic therapy (PDT) that also releases PL, blocking the TrxR‐mediated ROS elimination pathway.	[53]
	M/A@MOF@CM	mitoxantrone (MTO) and axitinib (AXB)	4T1 cells	Chemotherapy, photothermal therapy (PTT), and CDT, immunogenic cell death.	[54]
	CM‐MMNPs	MnO_2_	HeLa Cell	Enhancing O_2_‐mediated PDT effect.	[55]
RBC cell membrane	RBC@Mn‐MOF/PPI	polyphyllin I	RAW264.7, 4T1 cell	Committed to transforming immunologically “cold” tumors into “hot” tumors.	[56]
	PAB@MIL‐53 (PMNP) nanoparticles	PAB, DOX	MCF‐7 cells, MCF‐7/ADR cells	Active oxidants deplete glutathione and amplify oxidative stress.	[57]
	FA‐EM@GO‐MOF/DOX	GO, DOX	4T1 cell	PDT and PTT promote the production of tumor‐associated antigens and induce an anti‐tumor immune response.	[58]
	RGD‐mGZD	GOx, DOX	U87 cells, Hela cells	Preparation of a multifunctional bioreactor for synergistic starvation chemotherapy.	[59]
	O_2_@UiO‐66@ICG@RBC	oxygen	RAW264.7 cells	Elimination of hypoxic tumors by improving oxygen supply and circulating lifetime of photosensitizers.	[60]
	MA272@MOF@RBC	MA, CM272	Leukemia cells	Inhibited the growth of leukemia cells by targeting DNA and histone methylation while enhancing m6A‐RNA methylation.	[61]
	FTP@RBCM	FTP	Hep3B cells	Boosting radical storms to eradicate cancer cells in hypoxic environments.	[62]
Immune Cell Membrane	macrophage membrane‐coated PLNP@UiO‐66	doxycycline hydrochloride, acetylsalicylic acid, and paclitaxel)	SCC‐7 cells	Achieve sustained luminescence imaging‐guided drug delivery and tumor therapy without autofluorescence.	[63]
	MM@PIP@MIL‐100(Fe)	Piperine	MCF‐7, MDA, SKBR‐3, BT‐549, HaCaT cancer cells	Enhance immune system evasion and improve the efficacy of piperine in the treatment of breast cancer.	[64]
	MIL‐88‐MG132@M	MG132	CT‐26 cells	Promote the accumulation of pro‐apoptotic or misfolded proteins, disrupts tumor homeostasis.	[65]
	SonoCu	PFC,Ce6	4T1 cells.	Anticancer combination of SDT and cuproptosis.	[66]
	AP@ZIF‐Mem	ATO, PD	RAW 264.7 cells	Blocking glycolysis and associated pentose phosphorylation pathway disrupts the energy supply to tumors.	[67]
	mRDZ	DOX, siIDO1	RAW 264.7 cell, CT26 cells	Potently inhibit the growth, lung metastasis, and recurrence of tumors.	[68]
	PCoA@M	Anethole Trithione (ADT)	4T1 cell, C26 cell	Disrupting the balance of energy metabolism by consuming NADH to achieve tumor starvation therapy.	[69]
	AM@ZIF@NM	anti‐miRNA‐155	RAW 264.7 cells	Downregulated the expression of miR‐155 while rescuing the expression of its target gene BCL6.	[70]
	PmMN@Om&As	Oxymatrine, astragaloside IV	NIH3T3, CTLL‐2, H22 cells	Enhance the mitochondrial function of TILs by increasing their oxygen consumption rate and proton efflux rate.	[71]
	P‐MOF‐siRNA	siRNA	SK‐BR‐3 cells	Achieving effective gene silencing in vivo can expand the application of siRNA in various disease‐related contexts	[72]
Stem Cell membrane	TP‐M‐Cu‐MOF/siATP7a	/siATP7a	H69 cells, bEnd.3 cells	Activating gene silencing, copper efflux blockade, and lysine oxidase expression repression leading to cancer cell death.	[73]
	MOF‐DOX@DPSCM	DOX	CAL27 cells, FaDu cells, MCF7 cells	Against tumor growth through the implementation of CDT.	[74]
	AFMMB	azacitidine (AZA)	C1498 cells	Effectively target AML and successfully regulate STING and PD‐L1 by modulating DNA and RNA methylation.	[75]
	Fe_3_O_4_@PDA–siRNA@MSCs	siRNA	DU145 cells	Imaging‐guided PTT and gene therapy.	[76]
	PDA‐DOX/siPD‐L1@SCM	DOX, PD‐L1 siRNA	PC‐3 cells	Combined tumor chemotherapy and gene therapy.	[77]
Bacterial membrane	BMMZA@ERm	DOX and bortezomib (BTZ)	4T1 tumor cells	Construct a nano‐turret for chemotherapy‐assisted ascorbate‐mediated immunotherapy (CAMIT).	[78]
	CuM@RR	Cu‐based metal–organic framework (CuM)	4T1cells	Facilitate cuproptosis, PTT, and immunotherapy for the effective elimination of malignant solid tumors.	[79]

**SCHEME 1:**
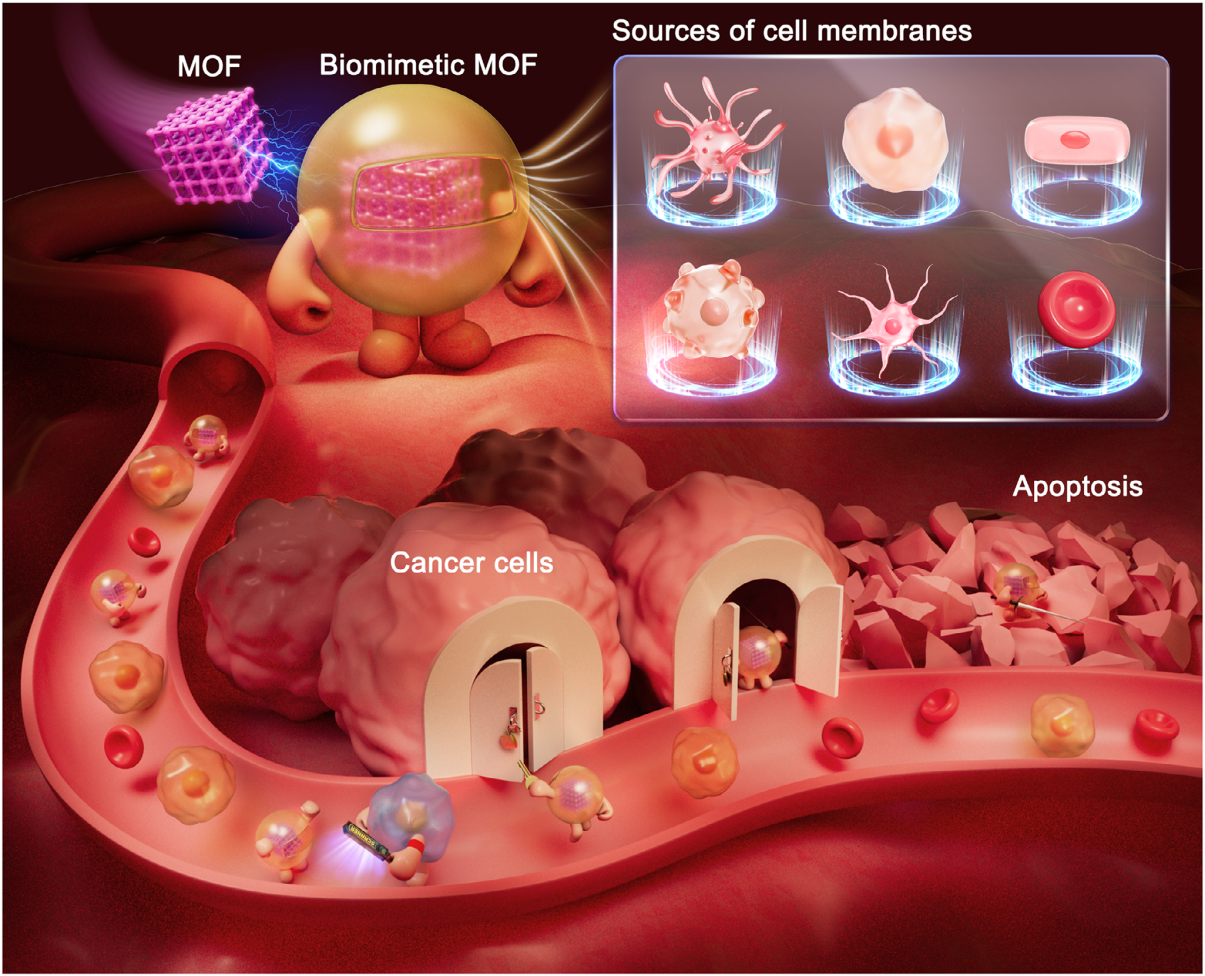
Biomimetic MOF‐based drug delivery nanoplatforms synthesized using liposomes or cell membranes as camouflage for multiple cancer therapeutics.

In this review, we focus on cell membrane‐camouflaged MOFs, with a view to enhancing drug delivery in various cancer therapeutic approaches. As shown in Scheme 1, systemic delivery of cell membrane‐coated MOFs is predominantly achieved via intravenous administration. Upon entering the systemic circulation, the biomimetic coating serves a dual function: it first prolongs vascular circulation time by evading immune clearance and subsequently facilitates trans endothelial migration or extravasation at the tumor site. This vascular exit is driven by a synergy of passive accumulation through the leaky tumor endothelium (EPR effect) and active interactions between membrane adhesion proteins and the inflamed vascular endothelial cells. This paper also summarizes key issues and challenges facing drug delivery systems in cancer therapy, namely targeting, stability, and biosafety. It further discusses how the development of biomimetic MOFs has led to new opportunities for cancer therapy. Biomimetic MOFs exhibit distinctive characteristics, including ligand diversity, structural stability, and biosafety, which are advantageous for drug delivery applications. This review aims to explore the potential of biomimetic MOFs in cancer therapy, focusing on various types of cell membrane biomimetic approaches and different roles of MOFs to enhance our understanding of biomimetic MOFs nanomaterials and to promote their development in the field of cancer therapy.

## 2 Cancer Cell Membrane‐Camouflaged MOFs

In recent years, the development of cell membrane biomimetic technology and MOFs has provided new approaches for nucleic acid delivery. Vesicles derived from cell membranes are widely used in delivery systems due to their excellent biocompatibility, immune evasion capabilities, and targeting recognition functions. Meanwhile, MOFs have become an ideal nanocarrier because of their highly tunable porous structure, exceptional loading capacity, and controllable degradability. Combining cell membranes with MOFs in delivery systems can achieve synergistic effects, addressing challenges such as stability, premature release of drugs and targeted delivery in vivo. Cancer cell membrane‐coated nanoparticles represent an emerging tumor‐targeting delivery strategy. Since cancer cell membranes contain the same proteins and antigens as the original tumor cells, they can interact with the same type of cancer cells, enabling self‐recognition, immune evasion, and efficient tumor targeting. This characteristic allows cancer cell membrane‐coated drugs to actively target the tumor microenvironment while minimizing the impact on healthy cells [80]. Specifically, this homotypic targeting property, which is hypothesized to be partially attributable to inherited membrane proteins, is the key factor of cancer cell membrane for cancer therapeutics [81]. Because it has been demonstrated that the presence of surface adhesion molecules on cancer cells that possess homologous adhesion domains can result in the formation of multicellular aggregates [82, 83]. Consequently, results indicate that drugs can accumulate in large amounts at tumor sites, reducing systemic toxicity.

**FIGURE 1:**
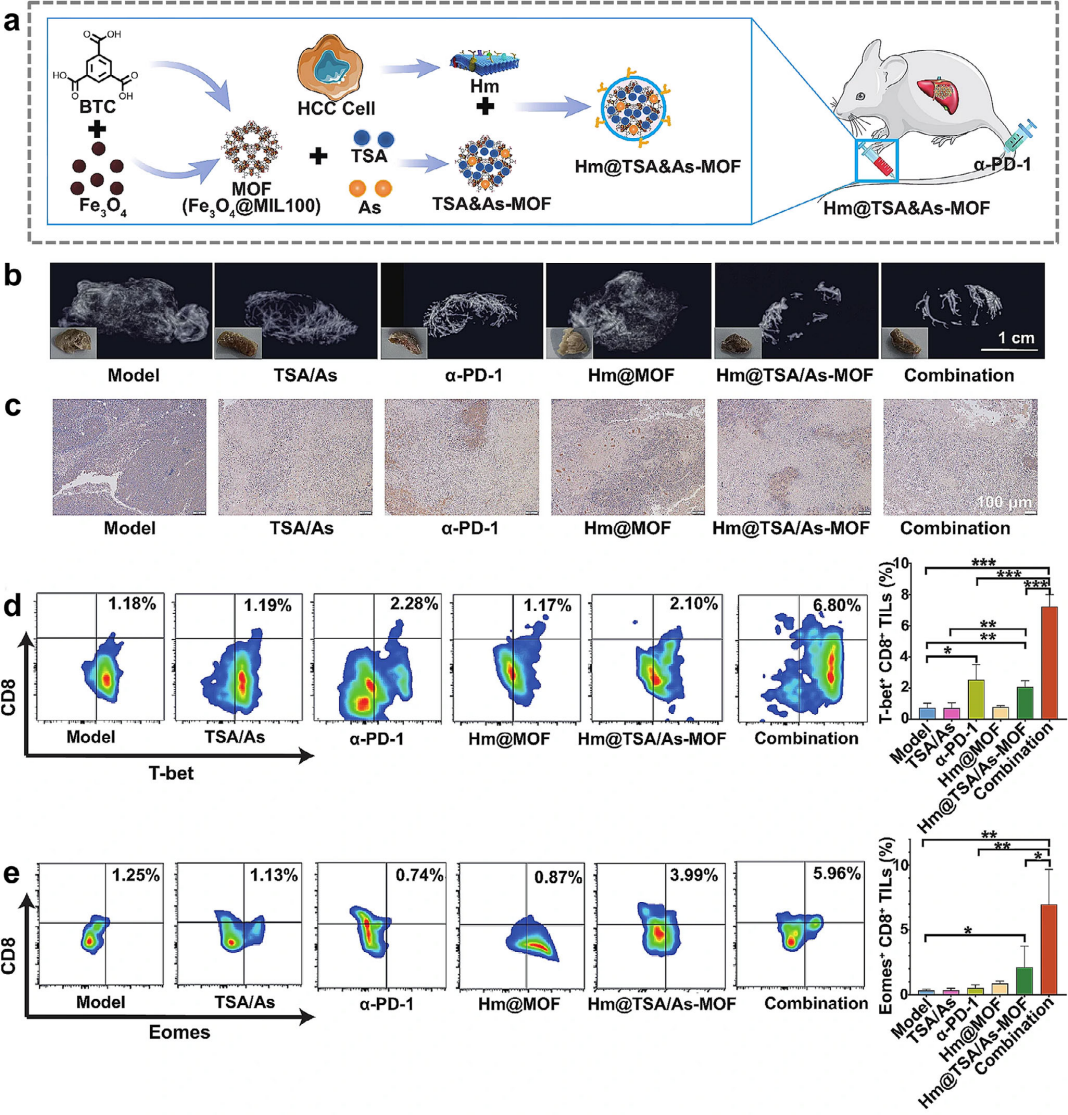
(a) Schematic illustration of the synthesis of biomimetic MOFs called Hm@TSA&As‐MOF which involved the use of Fe_3_O_4_ nanoparticles and BTC to form Fe_3_O_4_ @MIL101. Then, after post loading of TSA and As into the MOFs, homologous tumor cell membranes (Hm) were coated on the surface of MOFs (Hm@TSA&As‐MOF). (b) The levels of VEGF in tumor tissues (scale bar = 100 µm). (c) The pericyte coverage rate of tumor blood vessels (scale bar = 20 µm). (d) The proportion of T‐bet^+^ in CD8^+^ TILs. (e) The proportion of Eomes^+^ TILs in CD8^+^ TILs (n=3) [50]. Reproduced with permission. Copyright 2024, Springer Nature.

The homotypic targeting ability of cancer cell membranes for tumors, in combination with the use of MOF as a delivery platform, plays an important role in a variety of cancer therapeutics. Immunotherapy is a revolutionary cancer treatment technique that controls and eliminates tumors by restarting or restoring the anti‐tumor immune response within the body, and it has shown highly specific and efficient therapeutic effects on some specific types of tumors. Immunotherapies include immune checkpoint blockade (ICB), targeted antibodies, cancer vaccines, cell‐based therapies and immune cell engagers [84]. However, ICB is not for all types of cancer, and the objective response rate of PD‐1 inhibitors may not be more than 20% in clinical settings [85, 86] because inadequate level and activity of TILs in hypoxic tumors limit their anticancer efficacy [87]. So, combining ICB with other drugs or molecules that can increase the activity of TILs is an auspicious approach for cancer treatment. For example, oxymatrine (Om), a soluble alkaloid that inhibits the construction of mesenchymal barriers by cancer‐associated fibroblasts (CAFs), was effective in reducing the inhibition of intratumoral infiltration of TILs by mesenchymal barriers [88, 89]. Astragaloside IV (As), an insoluble tetracyclic triterpenoid, on the other hand, improves mitochondrial function in TILs [90, 91]. In addition, Tanshinone IIA (TSA), a diterpenoid, can also regulate vascular normalization and thus increase the number of TILs in the tumor vasculature [92]. Recently, the combined administration of TSA and As has proved that they can enhance the activity of TILs by vascular normalization and reducing the levels of immunosuppressive factors [50]. As shown in Figure 1a, utilizing Fe_3_O_4_@MIL100 as a delivery vehicle for both molecules can effectively improve their biocompatibility, and the homotypic cancer cell membrane wrapped around the outer layer can, in turn, effectively avoid the capture and removal by the mononuclear phagocyte system (MPS). The results showed that this strategy could effectively normalize tumor vasculature, reduce levels of immunosuppressive factors and augment the expression of pivotal regulators (T‐bet and Eomes) within CD8^+^ to amplify the anti‐tumor effects, thus improving the effectiveness of ICB (Figure 1b–e) [50].

**FIGURE 2:**
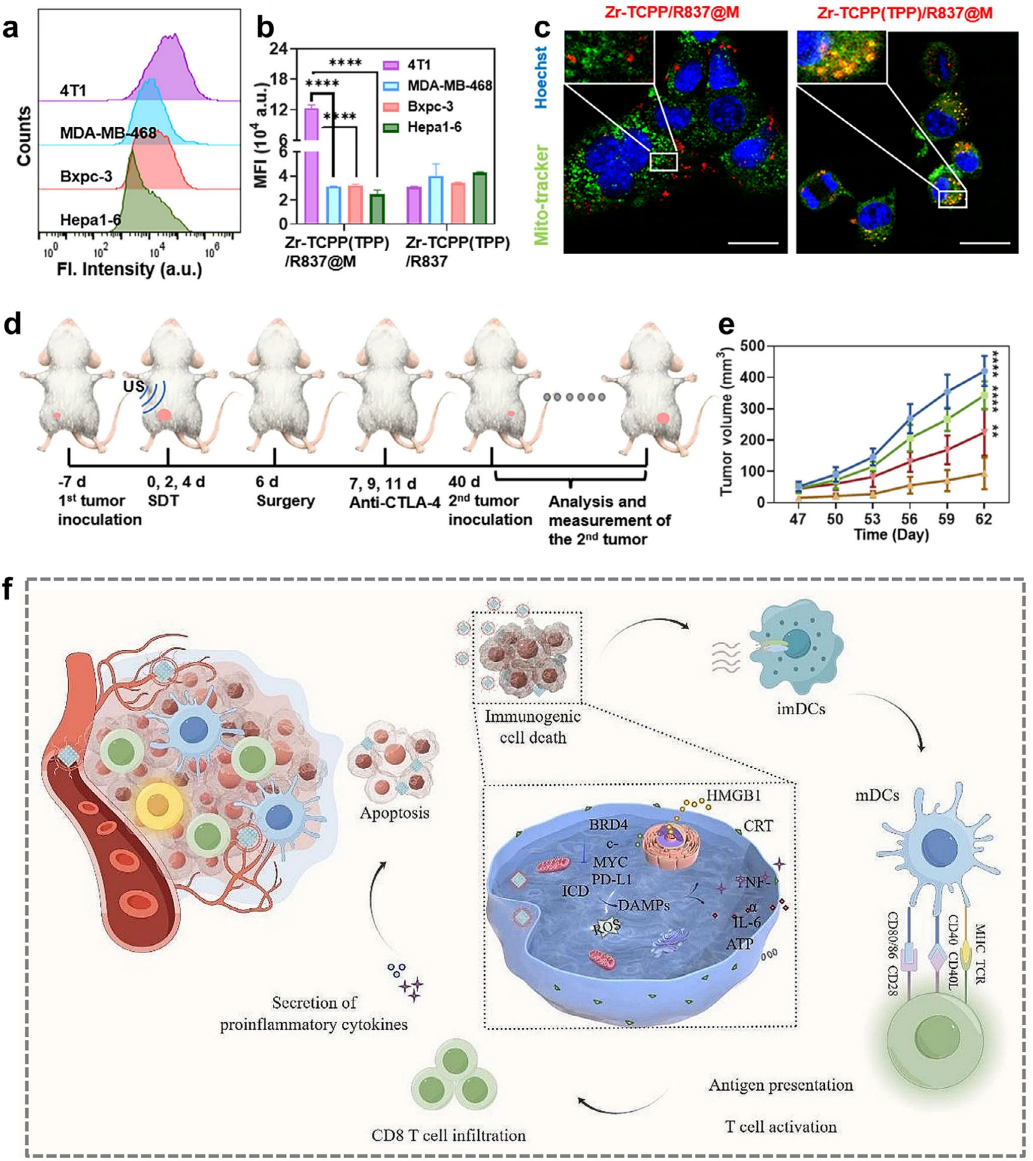
(a) Homotypic effect of Zr‐TCPP(TPP)/R837@M on 4T1, MDA‐MB‐468, Hepa1‐6, and Bxpc‐3 cells. (b) Statistical analysis of four different cell lines after treated with Zr‐TCPP(TPP)/R837@M or Zr‐TCPP(TPP)/R837 for 8 h. (c) CLSM images of subcellular localization of Zr‐TCPP(TPP)/R837@M, and Zr‐TCPP/R837@M. Scale bar: 25 µm. (d) Schematic diagram of experimental design for evaluating the anti‐tumor immune response and immune memory response against simulated distant tumors triggered by the Zr‐TCPP(TPP)/R837@M combination of enhanced SDT and anti‐CTLA‐4 to inhibit cancer recurrence. (e) The growth curves of the tumors in the revaccinated group (n = 5) exhibited a decline 40 days after the initial inoculation [46]. Reproduced with permission. Copyright 2022, BioMed Central. (f) Schematic diagram of the targeted therapeutic mechanism of MCM@UN in triple‐negative breast cancer [99]. Reproduced with permission. Copyright 2024, BioMed Central.

Moreover, cytotoxic T‐lymphocyte‐associated antigen 4 (CTLA‐4) is an important immune checkpoint, and the FDA has already approved its antibodies as a class of immunotherapeutic [93]. However, the lack of tumor‐associated antigen tumor‐associated antigens (TAAs) in the tumor immunogenicity and immunosuppressive microenvironment (TIME) and inadequate DC‐mediated antigen presentation have seriously affected the effectiveness of ICB [46]. Meanwhile, it was found that SDT not only stimulates immunogenic cell death (ICD) but also activates anticancer immune responses [94, 95]. Therefore, Luo et al. synthesized a cancer cell membrane‐coated triphenylphosphonium (TPP) decorated MOFs nanostructure [Zr‐TCPP(TPP)/R837@M] with a higher loading capacity of sonosensitizer and better targeting efficiency due to the large surface area and pore volume of MOFs and active targeting capability of the homologous cancer cell membrane, respectively (Figure 2a–c) [46]. The nanoplatform delivered sonosensitizer and Toll‐like receptor agonist R837 to enhance dendritic cell activity and combine it with anti‐CTLA‐4 to achieve more potent anticancer effects and develop durable anti‐tumor memory responses (Figure 2d,e) [46]. Similarly, for PD‐1 inhibitors, biomimetic MOF‐based SDT can likewise play an adjunctive therapeutic role and elicit more robust systemic immune responses and longer immunological memory function with anti‐PD‐1 antibody [52]. Taken together, due to the flexible, stable, biocompatible, active targeting and camouflage characteristics of biomimetic MOFs nanoplatforms, these nanomaterials can be effectively used as an adjuvant therapy for ICB which improves the therapeutic effect of immunotherapy in various ways.

Another problem of immunotherapy for cancer is the “cold” tumor with the absence or exclusion of T cells in the tumor parenchyma which makes them less sensitive to immunotherapy [96]. Converting immune “cold” tumors into “hot” tumors is a promising way to enable immunotherapy to have a higher response rate in cancer cells. The porous structure of MOFs provides excellent drug‐carrying conditions, among which UiO‐66 has become a popular candidate for drug delivery due to its excellent biocompatibility, stability, and pH responsiveness [97, 98]. At the same time, as a veneer of protection and camouflage, cancer cell membranes can also be therapeutic carriers. For example, Jiang et al. camouflaged TLR7/8 agonist in the cancer cell membrane and encapsulated BET inhibitor into UiO‐66 core to achieve a combined effect of ICB and epigenetic drugs for the treatment of triple‐negative breast cancer (TNBC), which is considered to be an immune “cold” tumor (Figure 2f) [99]. Herein, it is further demonstrated that such biomimetic MOF composites can be synthesized and modified to combine multiple therapeutic approaches in complex immunotherapy processes with the help of the material's unique characteristics.

**FIGURE 3:**
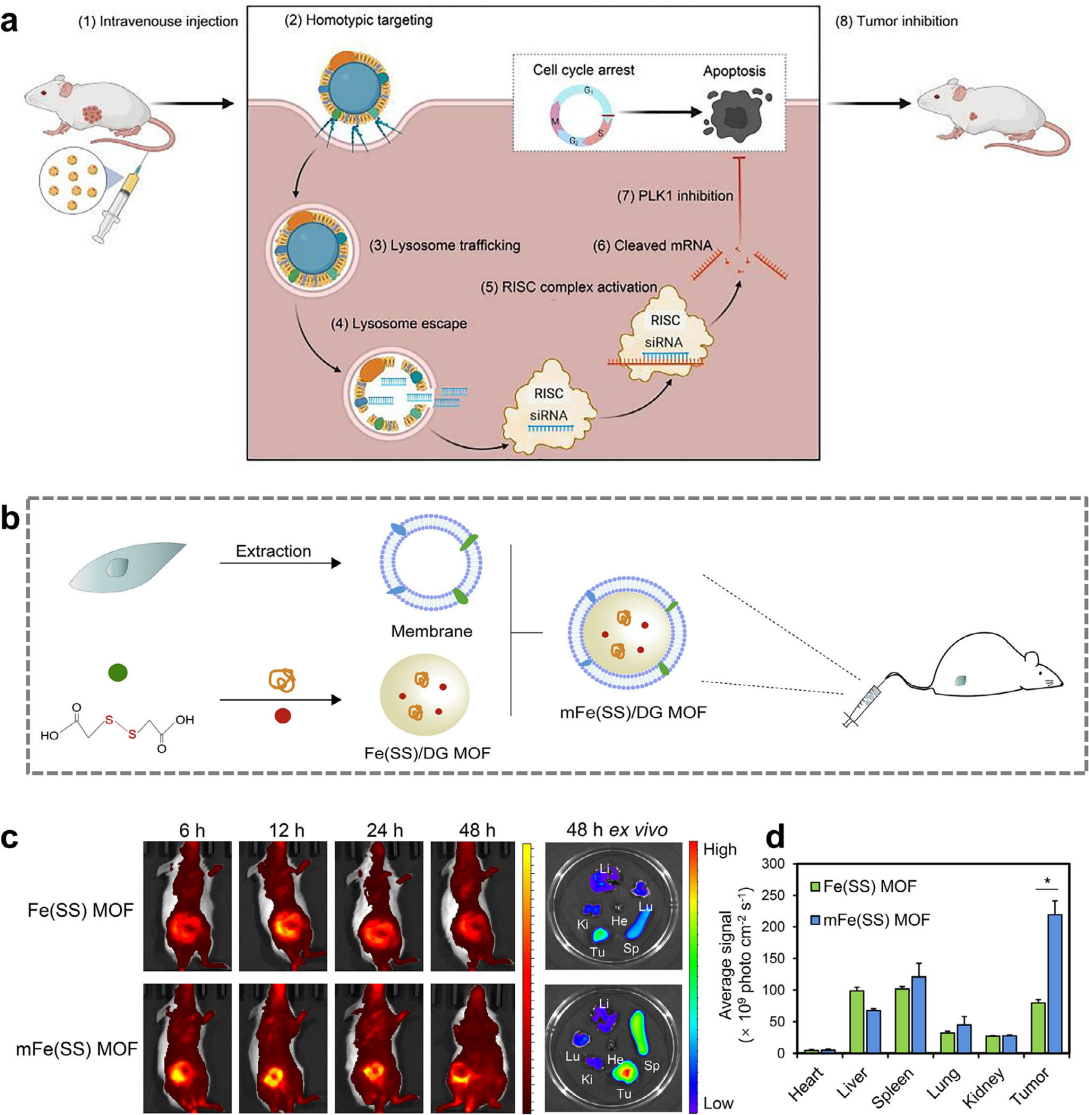
(a) Scheme illustration of the tumor inhibition effect and the systemic administrations of CAMEL‐R [47]. Reproduced with permission. Copyright 2021, Elsevier. (b) Schematic diagram of the construction of smart biomimetic nanoplatform mFe(SS)/DG MOF for antitumor chemoimmunotherapy. (c) The in vivo and ex vivo fluorescence images of 4T1 tumor bearing mice *i.v*. treated with DiR‐labeled Fe(SS)/DG and mFe(SS)/DG. (d) Semi‐quantification of the ex vivo fluorescence signals of the major organs and cancer tissues (n = 3). He, Li, Sp, Lu, Ki, and Tu stand for heart, liver, spleen, lung, kidney and tumor, respectively. Significance is defined as ns, no significance, *p < 0.05 [51]. Reproduced with permission. Copyright 2021, Elsevier.

Moreover, MOFs are a novel RNA delivery vector of interest because of their high surface areas, tunable structures, and high porosity to selectively bind and release RNA molecules through certain chemical modifications and fine‐tuning [100, 101, 102]. Several different interactions between nucleic acids and MOF materials have been found, such as binding the backbone of nucleic acids to metal sites or phosphate residues on the surface of the MOFs through covalent bonding [103, 104] or binding through strong electrostatic interactions [72, 105], in addition to binding through van der Waals forces [106]. The structural versatility of MOF materials improves the customization of delivery carriers, which can effectively protect fragile RNA molecules from damage in physiological environments, and MOFs can be used as a stimulus‐responsive material to release RNA molecules in a targeted manner, which improves therapeutic efficacy and reduces the damage to normal cells at the same time [47, 107, 108]. However, MOF‐based nucleic acid delivery systems still have fatal problems, such as insufficient circulation time in vivo and inability to aggregate at the target site, which hamper their application in cancer therapy. The zeolitic imidazolate framework‐8 (ZIF‐8) MOF, one of the most successful MOFs, has been widely used in many fields like drug delivery, biosensors, bone tissue engineering, photocatalyst and wastewater management [109, 110, 111, 112, 113]. The crystal topology of ZIF‐8 is similar to that of zeolites, and the resultant feature of connecting large cavities through small windows makes it very useful in biomolecular delivery [109, 114]. Zhang et al. used the Hela cancer cell membrane to camouflage the siRNA‐loading ZIF‐8 (CAMEL‐P) with high loading efficiency, large‐scale production, homotypic targeting ability and high anti‐tumor effect [47]. The nanoparticles coating with the cancer homotypic cell membrane significantly suppressed the abnormal expression of Plk1 in tumor cells. Specifically, the CAMEL‐P has also shown excellent biocompatibility, cellular uptake and lysosome escape ability because of the cell membranes and CAMEL‐P induced apoptosis in a large number of cancer cells (Figure 3a) [47]. Furthermore, another important factor of the CAMEL‐P, the circulation time, has been prolonged to the extent where about 30% siRNA still existed after 24 h, while nothing has been left for naked siRNA in vivo [47].

Another popular area of research in the treatment of cancer is CDT. CDT based on multiple different kinds of non‐apoptotic cell death has been found, such as ferroptosis, pyroptosis, and cuproptosis, which offers new opportunities for cancer treatment [115, 116]. Ferroptosis is recognized as an iron‐ and ROS‐dependent non‐apoptotic cell death pattern, in which iron accumulation, lipid peroxides (LPO) accumulation, and increased ROS are all key factors in its occurrence [117, 118, 119, 120]. Iron‐mediated oxidative damage and subsequent cell membrane damage underlie the onset of ferroptosis [118]. Normal cells regulate ferroptosis mainly through glutathione peroxidase 4 (GPX4) and glutathione (GSH) to avoid cellular damage [121]. Thus, ferroptosis can have a dual effect on tumor cells, possibly promoting or inhibiting. Fe‐based MOFs with cancer cell membrane coating are a suitable choice for the drug delivery system of ferroptosis‐based therapy because, as a highly efficient iron donor, their homotypic targeting ability and self‐contained iron ions can effectively stimulate the ferroptosis process to take place and load other drugs or molecules due to their porousness and high specific surface area, thereby providing synergistic treatment of cancer. Of course, combining Fe‐based MOFs with buthionine sulfoxide amine (BSO), a small molecule drug that inhibits GSH biosynthesis, can further enhance the effectiveness of ferroptosis [122]. Besides, Fe‐based MOFs can scavenge GSH based on the coordination between Fe^3+^ and disulfide‐bearing ligand therein, downregulating GPX4 levels and triggering ferroptosis [51]. Yang et al. synthesized a smart biomimetic nanoplatform mFe(SS)/DG MOF with Fe^3+^ as a metal node, dithiodiglycolic acid as an organic ligand, loaded with glucose oxidase (GOx) and Dox, and the outer layer was camouflaged by homotypic cancer cell membranes for more prolonged circulation and better‐targeting efficiency (Figure 3b–d) [51]. The presence of GOx as a trigger of starvation therapy not only catalyzed the production of large amounts of H_2_O_2_ from glucose, leading to the accumulation of excess ROS in the cell but also promoted ferroptosis and inhibiting glycolysis, thus enhancing the effect of chemotherapy and immunotherapy for cancer through ROS‐ferroptosis‐glycolysis regulation [51].

Furthermore, combining ferroptosis with other non‐apoptotic cell death could more effectively address the problem of cancer cells becoming resistant to apoptosis‐related drugs. Ji et al. have designed a multifunctional MOF nanoparticle which can kill cancer cells effectively with synergistic cuproptosis/ferroptosis/apoptosis anticancer therapy [116]. This nanostructure named M/A@MOF@CM consists of multifunctional Cu‐based MOFs loaded with MTO and AXB and an outer layer of tumor membrane cells [116]. The complex components and structure allow it to accumulate at tumor area and mimic the effects of chemotherapy, PTT and CDT, while studies have shown that the catabolism of M/A@MOF@CM requires the depletion of intracellular GSH and drives the onset of Fenton's reaction through the reduction of Cu^2+^, thereby decreasing the level of GSH while increasing the level of ROS to stimulate ferroptosis process [116].

**FIGURE 4:**
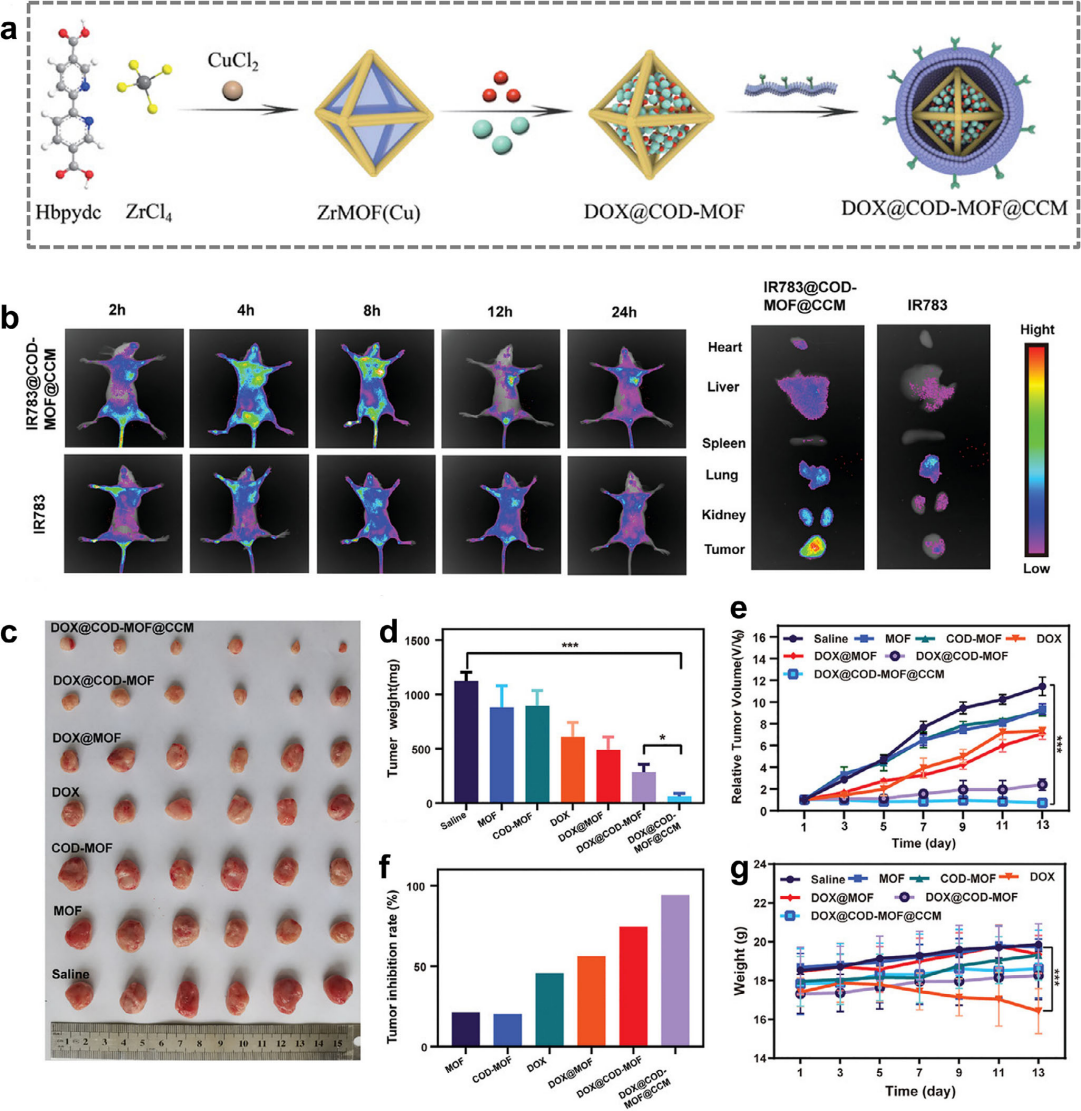
(a) Schematic diagram of the construction of the DOX@COD‐MOF@CCM. (b) Fluorescence imaging in BALB/c nude mice treated with IR783 and IR783@COD‐MOF@CCM nanoparticles, and the fluorescence imaging of the organs. (c) Tumor photos of the nude mice in different treatment method groups for 14 days. (d) Tumor weight of dissection with different treatment. (e) Tumor inhibition rate. f) Curve of relative tumor volume changes. (g) Weight of tumor bearing mice during treatment, ***p < 0.001 [44]. Reproduced with permission. Copyright 2022, Wiley.

In an ingenious advancement, the researchers integrated the targeting capability of cancer cell membranes with the responsive release mechanism of MOFs, thereby developing more effective chemotherapeutic agents. For example, ZIF‐8 is a pH‐sensitive material that degrades rapidly under acidic conditions, so relying on the characteristics of the microenvironment inside the tumor that is different from the normal cells, ZIF‐8 can also play a role in stimulus‐response release, thus achieving the purpose of targeting cancer cells [72]. As shown in Figure 4a, Guo et al. constructed a biomimetic “dual enzyme‐catalyzed cascade nanoreactor” (DOX@COD‐MOF@CCM) via Cu^2+^‐modified Zr‐based organic frameworks with cancer cell membrane encapsulation. This technology reduces the cholesterol content in cancer cells via cholesterol oxidase (COD), which reduces the membrane stiffness of drug‐resistant cancer cells and restores cellular sensitivity to DOX. Due to the advantage of homologous targeting, cancer cell membrane‐coated nanoreactors can accumulate well at the tumor site and inhibit 94.4% of tumor growth in vivo without systemic toxicity (Figure 4b–g) [44].

Meanwhile, the potential applications of biomimetic MOFs extend beyond their use as drug delivery platforms, encompassing their deployment as photosensitizer (PS) carriers or PS themselves within the domain of phototherapy. PTT and PDT are two main phototherapy strategies which rely on precise light irradiation targeting the tumor tissue, killing the cancer cells by thermal or chemical damage, respectively. However, phototherapies cannot fully meet the requirements for cancer treatment for now because the supply of O_2_ and tumor‐targeting capability are still limited [123, 124]. MOFs can make a great contribution as excellent gas adsorption materials for O_2_ delivery; some materials with the catalytic ability of O_2_ generation can also be combined with MOFs to form self‐supplying O_2_ nanoparticles. For example, Zhang et al. have constructed O_2_‐evolving PDT nanoparticles (CM‐MMNPs), which contain a MnO_2_ nanosheet on the surface of porphyrin‐based MOFs and encapsulates the membranes of cancer cells in the outermost layer [55]. In this hybrid nanostructure, based on porphyrin‐based MOFs working as PS, MnO_2_ gives the nanoparticles a highly sensitive H_2_O_2_ responsiveness in acidic solutions to produce O_2_, thus enhancing the O_2_‐mediated PDT effect [55].

An essential prerequisite for phototherapy is that the PS can be effectively accumulated at the target site. However, generally, most PS do not possess active targeting ability, which seriously affects the efficiency of the treatment and may also bring serious side effects. Hence, it is particularly important to improve the delivery efficiency to the target tumor cells and reduce the biotoxicity of PS. As mentioned earlier, using cell membranes as a coat for nanoparticles is an effective biomimetic technique for evading removal by the immune system in vivo [60]. It has been confirmed that circulating tumor cells have a strong affinity for each other, and the cell aggregation caused by this affinity is related to the recognition and interaction of proteins on the cell membranes [41]. Based on this result, researchers have proposed using isolated cancer cell membrane materials to camouflage nanoparticles to target homogeneous cancer cells. At the same time, this hybrid nanostructure has shown excellent stability, biocompatibility and targeting capability for tumor [55, 125]. For instance, Cheng et al. coated PCN‐PL (a porous MOFs of zirconium‐metalloporphyrin PCN‐222 loaded with alkaloid piperlongumine (PL)) with cancer cell membranes, and the results showed that PCN‐PL with CT‐26 cell membrane coating was more inclined to bind to CT‐26 tumor cells and entered into the cell interior, thus the cancer cell membrane coating endowed PCN‐PL with a particular function of homotypic cancer cell targeting [53]. Besides, Zhang et al. also demonstrated that the MOFs coated with HeLa cell membranes have a more pronounced preference for homologous cancer cells like HeLa and HepG2 cells compared to others [55]. Cancer cell membranes derived from melanoma have also been used to encapsulate Cu‐based MOFs for PTT of skin melanoma with enhancing tumor‐targeting abilities and immune response activation [126]. These results indicate that selecting the correct homotypic tumor cell membranes as coatings can effectively protect the MOF‐based PS encapsulated inside and enhance their active targeting capabilities to the targeted tumor cells, further improving the efficacy of phototherapies.

In summary, it can be found that the emergence of biomimetic MOFs not only provides an efficient delivery vehicle for traditional drug delivery, but also, after the emergence of a new cancer treatment approach, biomimetic MOFs can still play an important role and even combine a variety of traditional therapeutic means with new therapeutic mechanisms such as ferroptosis, to achieve excellent cancer therapeutic effects.

## 3 Blood Cell Membrane‐Camouflaged MOFs

Blood cells represent the most significant component of human blood, constituting approximately 45% of blood volume [127]. These cells can be categorized into three primary types: red blood cells, white blood cells, and platelets. In contrast to cancer cells, these blood cells lack homologous targeting capability for tumors, but their membranes carry molecules or proteins that are recognized by immune cells, which exempt them from being attacked by the immune system in vivo. Meanwhile, the inherent functionality of blood cells confers upon them certain advantages that other cells lack, such as the capacity to transport O_2_ and present antigens. Therefore, researchers extracted the cell membranes of blood cells and integrated them with MOFs, thereby further enhancing the development of biomimetic MOFs in cancer therapy.

### 3.1 Red Blood Cell Membranes

Tumor hypoxia is a common phenomenon due to abnormal vascularization, which cannot supply enough O_2_ and vital nutrients to the cells in solid tumors [128]. Meanwhile, hypoxia has a profound effect on the efficacy of anticancer therapies; these hypoxic or necrotic areas tend to be resistant to radiation, chemotherapy, and phototherapies and are strongly associated with poor prognosis in cancer patients [129]. Red blood cells (RBCs) also called erythrocytes are considered to be one of the most significant circulating cells in blood, as they contain an iron‐rich red protein, hemoglobin, which binds to O_2_, thus enabling RBCs to transport O_2_ from the lungs to the tissues [130]. In recent years, researchers have explored the potential of RBC to deliver O_2_ to improve hypoxia in tumor tissues. Concurrently, these cells and their membranes can function as effective carriers for nanomedicines, thereby enhancing the circulation time in the body and circumventing its immune system removal by the integral membrane protein CD47 releasing “don't eat me” signal [131, 132, 133, 134]. Furthermore, the presence of RBCs has been shown to induce alterations in pharmacokinetics (PK), with these changes exerting a significant influence on the reduction of drug dosage, enhancement of bioavailability, and mitigation of adverse effects. Of particular significance is the observation that RBCs exhibit “passive” delivery at pathological sites, including damaged vascular area, central nervous system, myocardial infarction area, and ischemic site [135]. Therefore, the use of RBCs and their cell membranes as outer camouflage for nanoparticles have become attractive.

**FIGURE 5:**
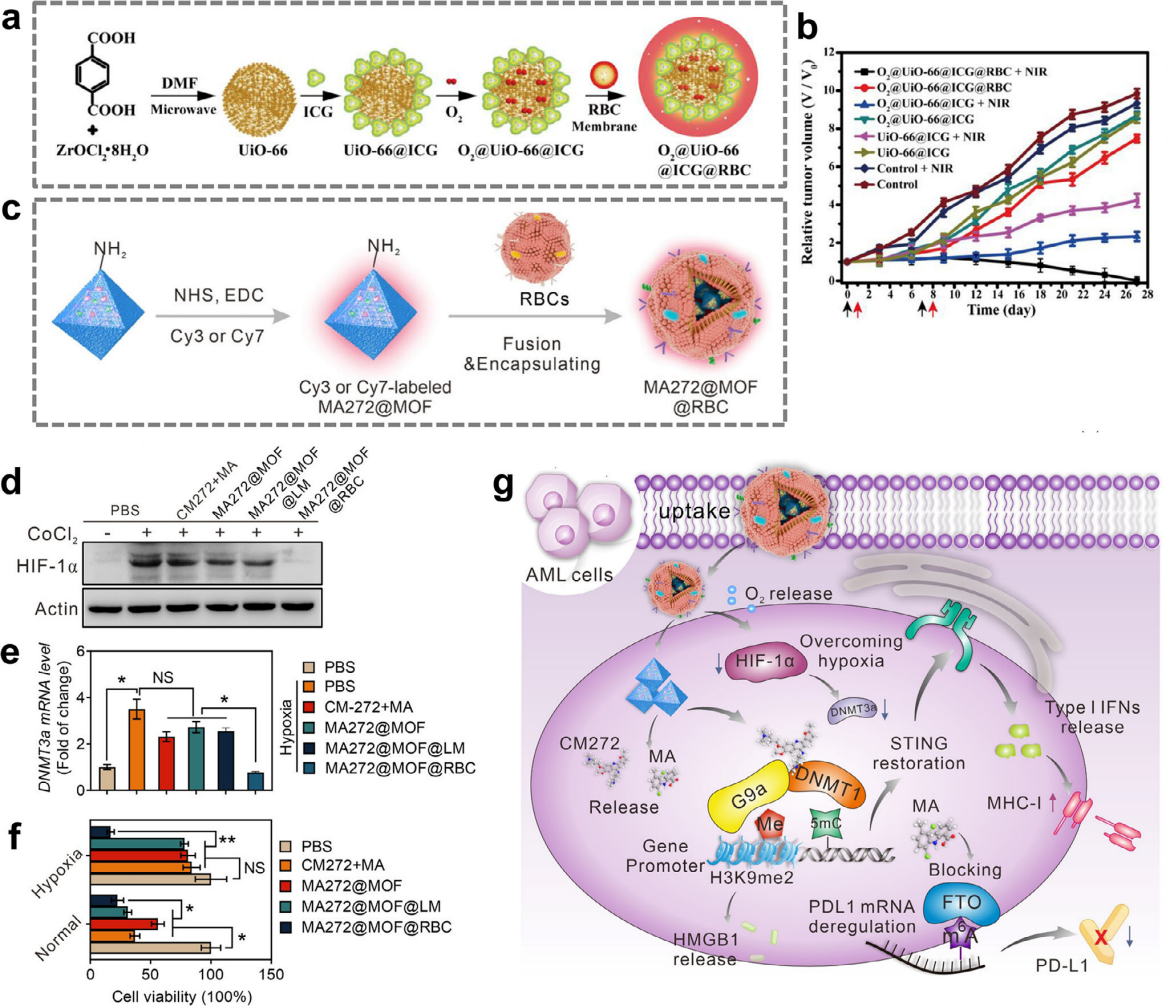
(a) Schematic diagram of preparation of O_2_@UiO‐66@ICG@RBC. (b) Relative tumor volume of MCF‐7 tumor‐bearing mice in different groups [60]. Reproduced with permission. Copyright 2018, Elsevier. (c) Schematic illustration of the synthesis of fluorescently labeled MA272@MOF@RBC. (d) The immunoblot analysis revealed the expression of HIF‐1α in C1498 cells treated with MA272@MOF@RBC or MA272@MOF@LM under CoCl_2_‐induced hypoxic conditions. (e) DNMT3a transcript levels. (f) Cell viability. *p < 0.05, **p < 0.01. (g) Schematic illustration of the triple epigenetic regulatory mechanism of MA272@MOF@RBC on AML cells [61]. Reproduced with permission. Copyright 2024, American Chemical Society.

As previously stated, the development of PDT and PTT continues to be constrained by O_2_ levels in tumor tissues and low targeting efficiency of photosensitizers. Hemoglobin can be encapsulated in MOFs coated with naturally derived RBC membranes as an effective O_2_ delivery system [136]. MOF‐based PDT nanoplatform with O_2_ delivering or generating ability can effectively increase the O_2_ concentration in the tumor microenvironment, relieving cancer cells from hypoxia, improving drug delivery and enhancing the efficiency of phototherapies. Luckily, the porous structures and high specific surface areas of MOFs give MOFs high PS or drug loading efficiency and a great gas storage capability [137], while combing with RBCs membranes has the potential to address the challenges associated with PDT and PTT in practical applications across multiple dimensions. Gao et al. synthesized a biomimetic O_2_‐evolving PDT nanoplatform O_2_@UiO‐66@ICG@RBC based on previous studies about UiO‐66 (zirconium (IV)‐based MOF) on O_2_ storage (Figure 5a) [60, 138]. Specifically, they used UiO‐66 as an essential carrier in the delivery platform, not only carrying the indocyanine green (ICG, a kind of PS which can be activated under 808 nm laser irradiation) but also adsorbing O_2_ in it, with the RBC membrane acting as an outer camouflage for the nanoparticles to evade the immune response, enhance the circulation lifetime of the nanoparticles, enhance the EPR (Enhanced Permeability and Retention) effect in vivo and achieve passive targeting for cancer cells. The results show that this nanoplatform can overcome tumor hypoxia, which exhibited excellent O_2_ self‐sufficient PDT effect (Figure 5b) [60]. Tumor hypoxia also exerts a significant impact on the efficacy of immunotherapy for cancer. For instance, hypoxia has been identified as a crucial marker in leukemia, where it activates hypoxia‐inducible factor (HIF‐1α) and genes associated with drug resistance [139]. Therefore, to address the significant issue of tumor hypoxia, Ding et al. constructed a UiO‐66‐NH_2_based erythrocyte biomimetic nanoplatform (MA272@MOF@RBC) which is shown in Figure 5c [61]. In this nanoplatform, RBCs not only play their role in aggregating at the tumor site, but also, because they still carry intracellular hemoglobin, deliver O_2_ along with the MOF, greatly reducing the hypoxia at the tumor site and enhancing the efficacy of the epigenetic drugs carried by the MOF. Meanwhile, the RBCs within the nanoplatform effectively downregulated the expression of HIF‐1α and further inhibited the transcription of DNA methyltransferase 3α (DNMT3a), a downstream gene regulated by HIF‐1α (Figure 5d–f). As shown in Figure 5g, the multifunctionality of RBCs effectively mitigated the hypoxia present within the leukemia tumor microenvironment, thereby significantly enhancing the therapeutic efficacy of MA272@MOF@RBC against cancer cells both in vitro and in vivo [61]. Besides, MOFs can not only be used as a carrier for PS and O_2_ [60], but the material itself can also play the role of PS in participating in the therapeutic process [53, 55, 140]. Meanwhile, the inhibition of antioxidants by MOF‐loaded drugs can also effectively circumvent the tumoral hypoxia problem to increase ROS levels and disrupt redox balance [53].

It is evident that the surface regulation and modification of RBC membranes themselves is also critical for camouflaging and delivering MOFs and anti‐tumor drugs. It has been determined that a reduction in cholesterol levels within the membranes of RBCs can effectively enhance the permeability of cell membranes. This increased permeability can serve to camouflage MOFs nanoparticles, while concurrently increasing the level of enzymatic activity of enzymes that are loaded in MOFs [141]. This outcome is particularly advantageous for anti‐tumor therapeutics involving enzyme catalysis, such as the catalytic generation of intracellular ROS. Furthermore, the modification of the tumor‐targeting ligand (RGD) on RBC membranes can enhance their targeting efficiency to tumor cells, thereby amplifying the anticancer efficacy of the biomimetic MOF nanomaterials [59]. In a recent study, researchers pioneered a novel approach by fusing RBC membranes with platelet membranes and subsequently wrapping them around MOFs nanoparticles [142]. This innovative strategy was found to result in the biomimetic MOFs demonstrating enhanced circulation times, augmented immune escape ability, and superior tumor cell targeting capabilities. While the underlying mechanisms remain to be elucidated, a hypothesis can be proposed. It is conceivable that both of the most common cell types in the blood stream express immune‐regulating molecules and membrane proteins which are not identical in kind, thereby rendering them more effective than single cell membranes in escaping from the clearance of immune cells. Given that these two cells have different but closely related functions in their natural environments, the membranes of the resulting fusion cells combine features of both, thereby enhancing the ability of biomimetic MOFs to deliver drugs in vivo.

**FIGURE 6:**
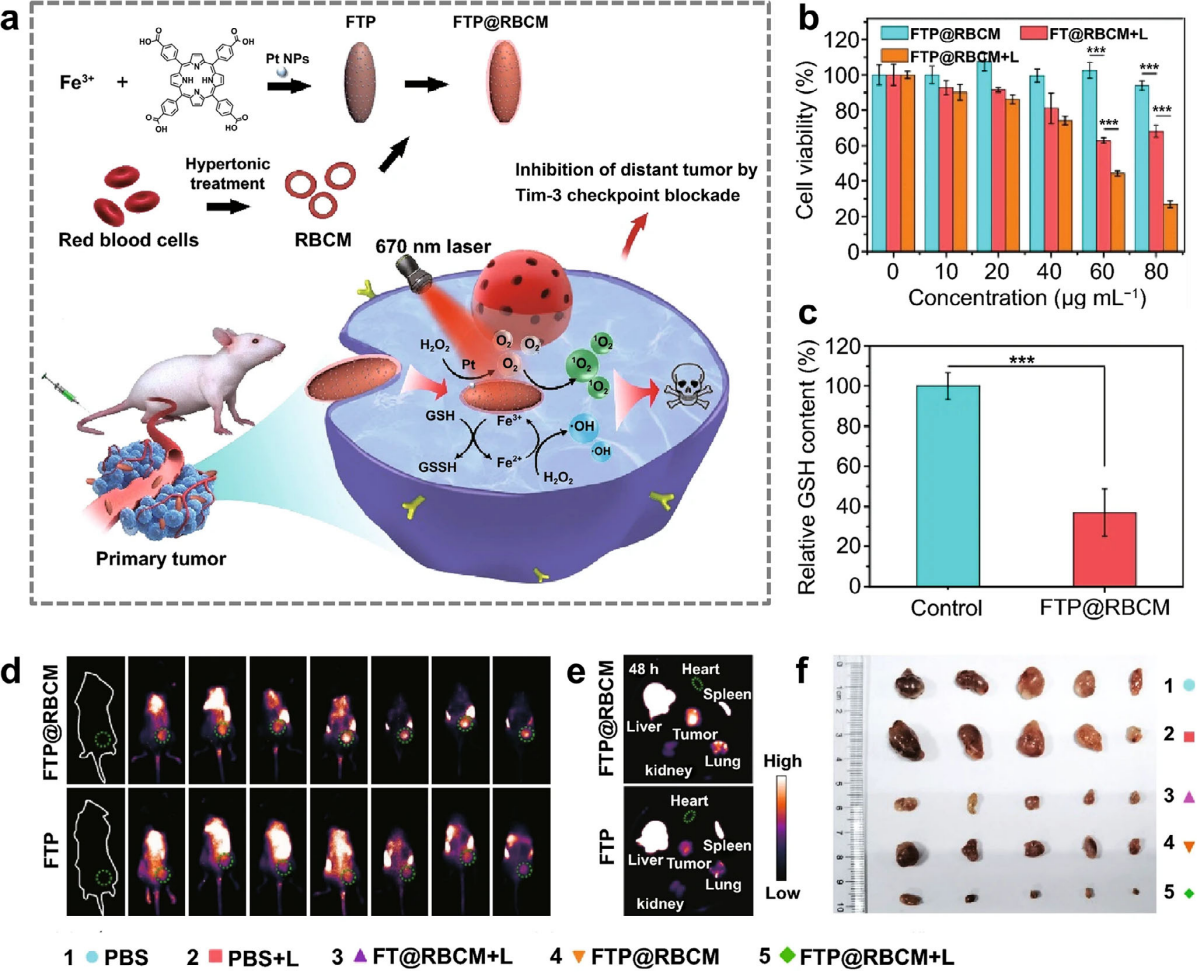
(a) Schematic illustration of the construction of artificial RBCs (FTP@RBCM) and immunotherapeutic mechanisms. (b) Cell viabilities of Hep3B cells under hypoxia. (c) Relative GSH content of Hep3B cells. (d) Fluorescence images of mice treated with ICG labeled FTP or FTP@RBCM. (e) Fluorescence images of organs and tumors. (f) Ex vivo tumor photos of mice [62]. Reproduced with permission. Copyright 2022, Springer Nature.

While ensuring the provision of O_2_ delivery, erythrocytes also have an immunological surveillance function, where they eliminate foreign pathogens through the production of ROS, a phenomenon that could theoretically be applied to the radical therapies of cancer [143]. Unfortunately, the production of ROS is limited and not sufficient to kill cancer cells. Nevertheless, the immunological surveillance capability of RBCs continues to motivate researchers to engineer a biomimetic RBC by integrating RBC membranes with particular MOFs to augment ROS production, thereby targeting cancer cells for anti‐tumor effects. For example, as shown in Figure 6a, Li et al. constructed a multifunctional artificial RBCs with Pt nanoparticles loaded Fe‐porphyrin‐based MOFs core inside and RBC membranes layer outside (FTP@RBCM) [62]. The utilization of biomimetic RBCs has been demonstrated to circumvent the limitations associated with natural RBCs, including fragility and suboptimal functionality. These biomimetic RBCs exhibit the capacity for long‐term circulation in vivo and targeted delivery to cancer cells (Figure 6d,e), while concurrently enhancing stability and flexibility of the nanocomposites. Upon entering the cancer cells, Fe‐porphyrin‐based MOFs, which contain platinum (Pt) nanoparticles, would be activated by 670 nm laser. These MOFs act as catalysts, facilitating a series of catalytic reactions, where GSH and H_2_O_2_ are consumed and converted into ROS resulting in an enhancement in the production of intracellular ROS (Figure 6c). The multifaceted functionality of O_2_ self‐supply, PDT, CDT, and glutathione‐like peroxidase catalytic activity, is a hallmark of the artificial RBCs which can operate by boosting radical storms to eradicate cancer cells in hypoxic environments (Figure 6b–f) [62].

### 3.2 Immune Cell Membranes

By camouflaging synthetic MOF cores with membranes derived from specialized immune cells, most notably macrophages for their inflammation‐homing abilities and platelets for their tumor‐targeting and immune‐evasion properties. Researchers have engineered a sophisticated class of “biological‐synthetic” hybrids capable of navigating complex physiological barriers with unprecedented precision.

**FIGURE 7:**
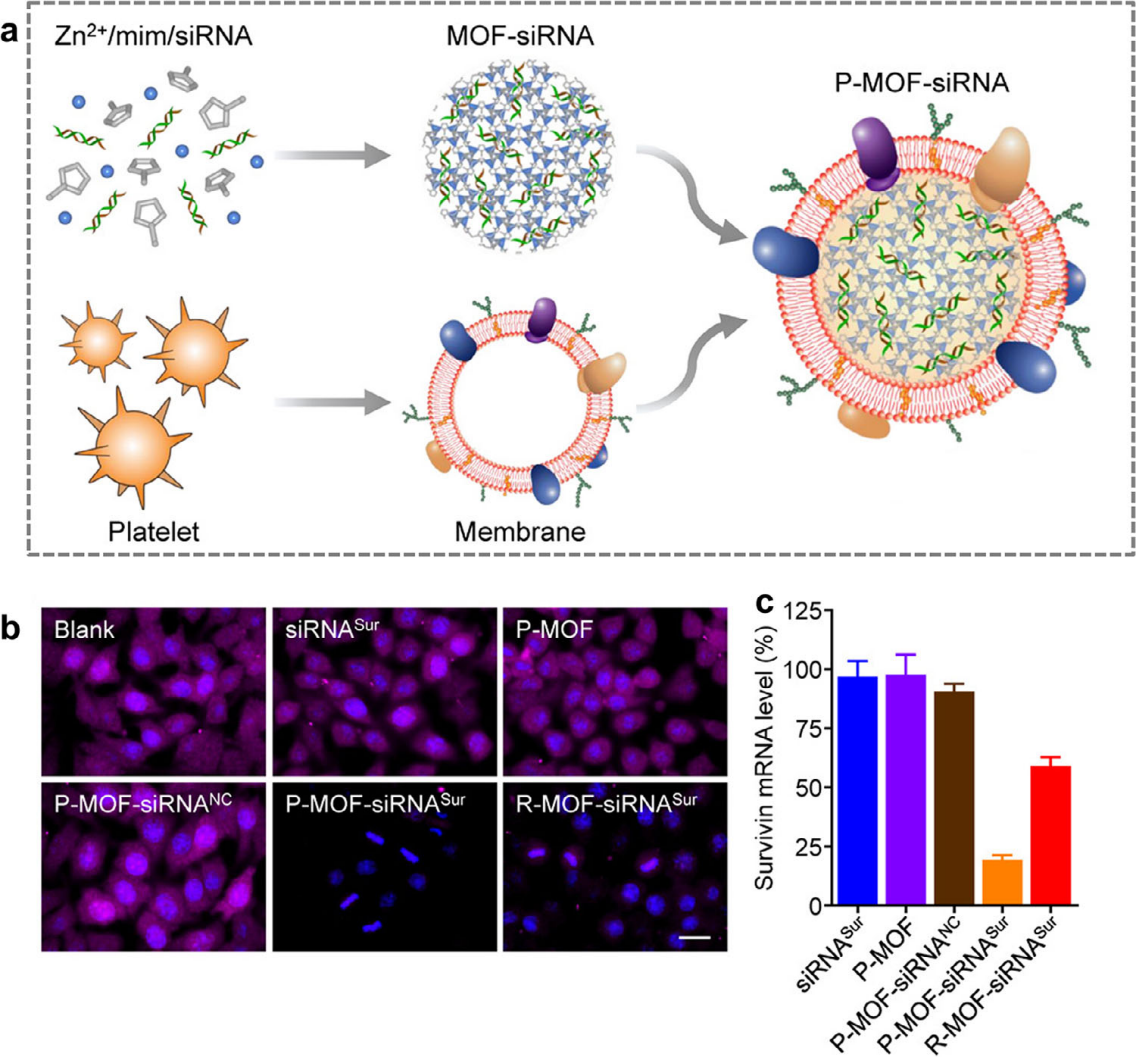
(a) The construction of platelet membrane–coated siRNA‐loaded MOFs (P‐MOF‐siRNA). (b) Fluorescent imaging of survivin protein expression in SK‐BR‐3 cells after incubation with different treatment for 48 h (scale bar, 20 µm; survivin, purple; nuclei, blue). (c) Relative survivin mRNA expression in SK‐BR‐3 cells after incubation with different treatment for 48 h (n = 3, mean + SD) [72]. Reproduced with permission. Copyright 2020, American Association for the Advancement of Science.

Platelets are a crucial blood component, playing critical roles in hemostasis, wound healing, and immune responses. The platelet membrane contains various immune‐regulating molecules and proteins, such as CD47, P‐selectin, and PF4, which effectively help avoid recognition and attack by the host's immune system [144]. Tumor microenvironments are often associated with abnormal vasculature and inflammatory responses, and platelets tend to accumulate at sites of inflammation, wounds, or tumors [145]. Moreover, immune cells, such as macrophages, T cells, and natural killer (NK) cells, possess the ability to recognize and respond to pathogens, damaged cells, and cancer cells. Like platelets, immune cell membranes express surface molecules such as CD47, enabling them to evade clearance by macrophages and other immune cells [146]. Zhuang et al. developed a biomimetic approach to deliver siRNA into cancer cells for therapeutic purposes [72]. ZIF‐8, known for its low toxicity and ability to release drugs in low pH environments, was selected to encapsulate siRNA. The siRNA‐loaded MOFs were then coated with naturally extracted platelet membranes, forming a platelet membrane‐coated siRNA‐loaded MOFs (P‐MOF‐siRNA) system (Figure 7a). When P‐MOF‐siRNA nanoparticles were endocytosed by target cells, the low pH environment inside the cells triggered the release of siRNA from the MOFs into the cytoplasm. Previous studies found that knocking down survivin mRNA in HER2‐positive SK‐BR‐3 cells could induce apoptosis [147]. To test the gene silencing effect of P‐MOF‐siRNA, the system was delivered to SK‐BR‐3 cells, and PCR data showed an 80% reduction in survivin mRNA levels (Figure 7b,c). Additionally, experimental results demonstrated that P‐MOF‐siRNA protected the siRNA in the presence of RNases [72]. This indicates that the platelet membrane not only effectively targets cancer cells but also protects RNA‐based therapeutics. Moreover, in immunotherapy, due to the poor bioavailability and biodistribution of Om and As, Guo et al. established a nanoplatform based on Fe_3_O_4_@MIL100 magnetic MOFs, which were coated by platelet membranes and loaded with Om and As. They combined this nanoplatform with α‐PD‐1 to improve anti‐hepatocellular carcinoma (HCC) efficacy, and their results have shown that tumor suppression rate (TSR) has reached 84.15% and the survival time of HCC‐bearing mice has been extended [71].

Macrophages are a core component of the immune system, widely distributed throughout body tissues, where they play multifaceted roles in immune defense, tissue homeostasis, and injury repair [148]. Macrophage membrane expresses pattern recognition receptors (e.g., TLRs), chemokine receptors, and antigen‐presenting molecules, which enable the active recognition and phagocytosis of pathogens as well as the clearance of apoptotic cells, while also activating adaptive immunity via the secretion of cytokines (such as TNF‐α and IL‐6) and antigen presentation [148]. The encapsulation of nanomaterials with macrophage membranes has emerged as a significant breakthrough in biomimetic nanomedicine in recent years. This approach offers three main advantages: natural immune evasion, precise targeted delivery, and high biocompatibility.

**FIGURE 8:**
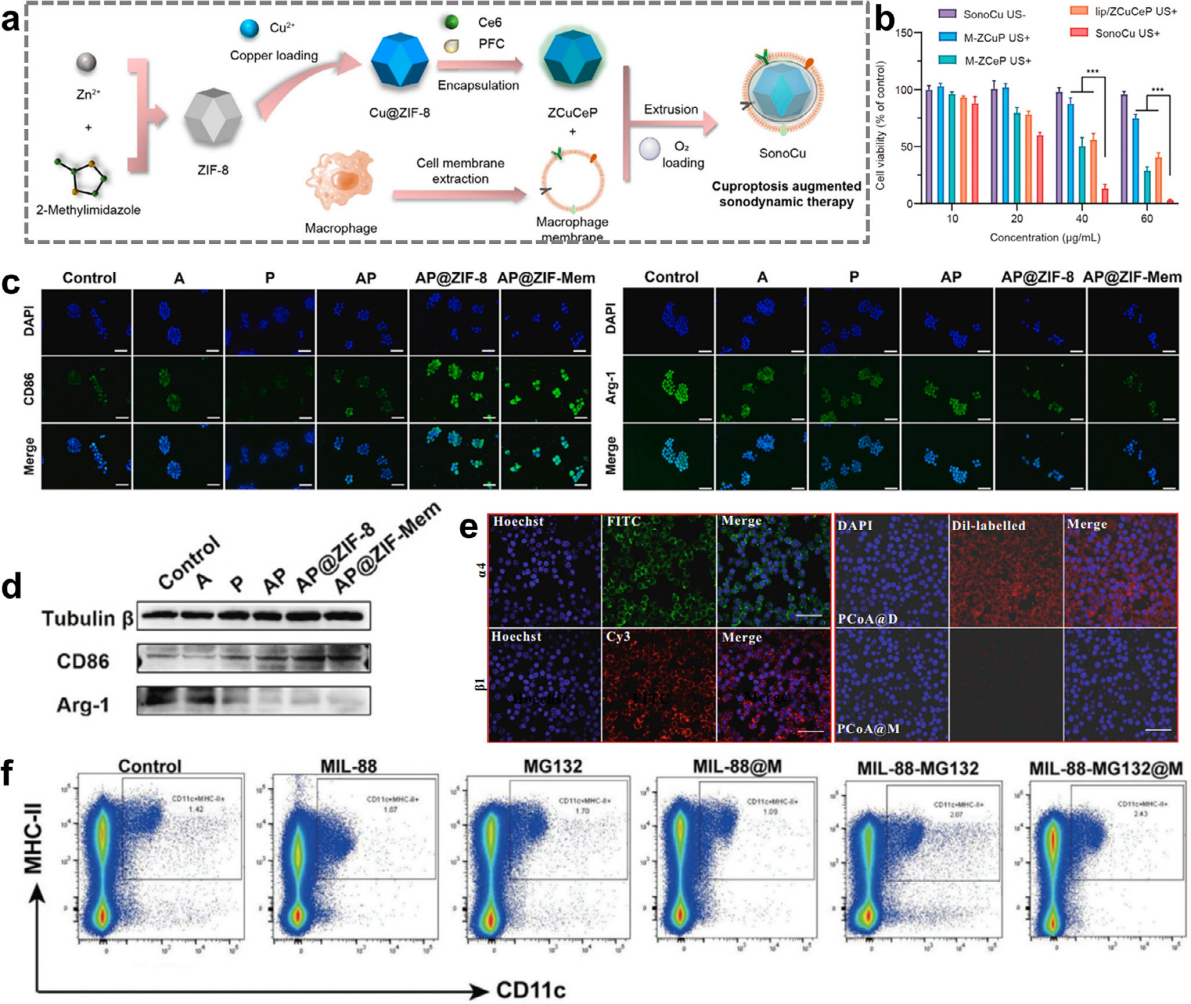
(a) Preparation of cell‐derived SonoCu. (b) In vitro anticancer effects of SonoCu against 4T1 cancer cells under normoxia [66]. Reproduced with permission. Copyright 2023, American Chemical Society. (c) The expression of CD86 and Arg‐1 on RAW264.7 cells after various treatments. (d) Western Blots showed expression of CD86 and Arg‐1 in RAW264.7 cells under different treatments [67]. Reproduced with permission. Copyright 2024, Elsevier. (e) Immunofluorescence imaging for the detection of α4 and β1 antigens on the macrophage membrane and Dil‐labelled PCoA@M and PCoA@D incubated with RAW 264.7 cells for 5 h [69]. Reproduced with permission. Copyright 2022, Springer Nature. (f) Expression levels of CD11c+ / MHC‐II+ [155]. Reproduced with permission. Copyright 2023, Wiley.

Studies have shown that cuproptosis, a copper‐dependent non‐apoptotic cell death involving alterations in the tricarboxylic acid (TCA) cycle pathway, is rapidly emerging as a promising cancer treatment strategy due to its unique advantages in combating different types of tumors [149]. Chen et al. developed an intelligent cell‐derived nanorobot (termed SonoCu) for improving cuproptosis augmented SDT. SonoCu was composed of macrophage membrane camouflaged nanocarrier encapsulating copper‐doped zeolitic imidazolate framework‐8 (Cu@ZIF‐8) (Figure 8a). Copper ions released from Cu@ZIF‐8 further amplified the efficacy of SDT by interfering with the TCA cycle, inducing lipid acylated protein aggregation and loss of iron‐sulfur cluster proteins, contributing to the accumulation of ROS, proteotoxic stress and metabolic regulation further amplifying the tumor‐killing power [66]. Macrophages have good tumor‐targeting capacity due to integrin α4‐VCAM‐1 interactions between macrophages and tumor cells [150]. Thus, MCM modification confers a longer systemic circulation time and active tumor targeting ability to SonoCu. Flow cytometry data and confocal laser scanning microscopy (CLSM) images showed that SonoCu accumulates rapidly in cancer cells, producing fluorescence intensities that are 1.80 times higher than lip/ZCuCeP (Figure 8b). Macrophage membrane artifacts significantly reduced the immunogenicity of SonoCu. In vivo experiments showed no pathological damage to major organs and normal serum liver and renal function indices [66].

The metabolic characteristics of cancer cells have been well exposed to be significantly different from those of normal cells, the most prominent of which is the Warburg effect. Abnormal glucose metabolism promotes a microenvironment for tumor growth, allowing tumor cells to escape the normal process of apoptosis and promoting proliferation and migration [151]. Thus, the inhibition of glycolysis and lactate production or secretion in tumor cells has become a strategy for cancer therapy. To interfere with glucose metabolism in tumor cells, the researchers developed metal‐organic framework nanoparticles (AP@ZIF‐Mem) that encapsulate macrophage membranes [67]. Atorvastatin and doppler were chosen as drugs to inhibit the lactate transporter MCT4 during glycolysis and the rate‐limiting enzyme G6PD in the pentose phosphate pathway (PPD). This two‐pronged intervention not only interferes with glucose metabolism but also modulates the tumor's immunosuppressive microenvironment. Breast cancer is the most diagnosed cancer in women and the leading cause of cancer deaths in women. During metastasis of triple‐negative breast cancer, angiogenesis is promoted by the massive secretion of vascular cell adhesion molecule‐1 (VCAM‐1). Fortunately, high expression of integrin‐α4 in macrophages promotes interaction with VCAM‐1 on tumor cells, enabling active targeting. The results of immunofluorescence detection assay (Figure 8c) and Western Blot (Figure 8d) showed that AP@ZIF‐Mem over immunofluorescence assay indicated that macrophages were successfully transformed from M2 to M1 phenotype in the microenvironment of tumor cells treated with AP@ZIF‐Mem, enhancing anti‐tumor immunity. In addition, zeta potential test of nanoparticles showed that the surface potential of AP@ZIF‐8 was 13.6mv, whereas after being wrapped by macrophage membrane, the surface potential of AP@ZIF‐8 decreased to about −19.85mv, which is close to the natural cell membrane charge [67].These data suggest that macrophage membrane able to reduce immune recognition and improve tumor targeting.

Indoleamine 2,3‐dioxygenase 1 (IDO1) plays an important role in malignant tumors of epithelial origin, such as colorectal cancer. IDO1 inhibits ROS production and autophagic response, which contributes to the growth of cancer cells [152]. Inhibition of IDO1 activity may be a key approach to reactivate anti‐tumor immune responses and remodel the immunosuppressive tumor microenvironment. Unfortunately, monotherapy with IDO1 inhibitors has not been effective in clinical trials. Zhou et al. utilized the advantage of ZIF‐8 as a carrier to construct mRDZ NPs by incorporating DOX and siIDO1 into ZIF‐8 nanoparticles, which were then encapsulated with macrophage membrane biosurfaces, thus realizing a multi‐pathway immunomodulatory cancer treatment strategy with the synergistic effects of ICD, autophagy and IDO1 [68]. mRDZ successfully established local and systemic anti‐tumor immune memory, results showed that immune memory T cells were also maximally activated and expanded after mRDZ treatment. In addition, experimental data also demonstrated that macrophage‐encapsulated mRDZ NPs exhibited better tumor‐targeting ability in vivo [68]. When comparing the fluorescence intensity of DOX in tumor tissues between different groups, the fluorescence intensity of the mRDZ group was 6.31‐fold fluorescence intensity of the free DOX group and 1.90‐fold fluorescence intensity of the RDZ group [68]. In summary, mRDZ proved to be able to effectively inhibit tumor growth, lung metastasis and recurrence, and can be used as a powerful anti‐tumor immune remodeling tool for the design of nanomaterials.

Heat shock protein 90 (HSP90) is an important molecular chaperone protein that is widely found in the human body, but then some cancer tissues such as hepatocellular carcinoma, colon cancer, etc. contain significantly higher levels of it than normal tissues [153]. Therefore, HSP90 is also used as a promising tumor biomarker. Cheng et al. constructed a biomimetic multifunctional nanoplatform PCoA@M based on macrophage membranes, and this nanoparticle contains polydopamine (PDA), cobalt‐based metal‐organic framework (Co‐MOF) and ADT. PDA, as the core of PTT, has efficient photothermal conversion ability, while Co‐MOF releases Co^2^^+^ to inhibit the expression of HSP90 and reduce the thermotolerance of tumor cells, which enhances the PTT effect. ADT induces tumor cell apoptosis by enzymatically interpreting the release of high concentration of H_2_S, while disrupting the balance of energy metabolism by consuming NADH to achieve tumor starvation therapy. The immunofluorescence assay results showed that the α4 integrin secondary antibody emitted strong green fluorescence and the β1 integrin secondary antibody also emitted red fluorescence (Figure 8e). These data suggest that many α4 and β1 integrin proteins are expressed on the membrane surface of macrophages. Therefore, combining VCAM‐1, a vascular cell adhesion molecule on tumor cells, with integrin proteins, the tumor‐targeting recognition effect of macrophage membranes could be achieved. In addition, they also demonstrated that probes modified with macrophage membranes produce good camouflage effects, thereby reducing phagocytosis by macrophages [69].

Epigenetic regulatory mechanisms include DNA methylation, histone modification and non‐coding RNA regulation [154]. Abnormal epigenetic regulation is believed to play a key role in the development of cancer. Through sequential modulation of ubiquitination and phosphorylation epigenetic mechanisms, Bu et al. developed a Fe‐MOF‐based nanoplatform (MIL‐88‐MG132@M), which enhances the efficacy of CDT in metastatic colorectal cancer (mCRC). In the course of CDT, MIL‐88‐MG132@M catalyzes the generation of hydroxyl radicals (‐OH) from H_2_O_2_ in the tumor microenvironment (TME) via the Fenton reaction, triggering oxidative stress to kill tumor cells. In contrast, the encapsulated MG132 blocks the 26S proteasome, promotes the accumulation of proapoptotic or misfolded proteins, disrupts tumor homeostasis, and downregulates driver gene expression. In addition, an activated immune response is partly responsible for mCRC recession. In treatment utilizing MIL‐88‐MG132@M, flow cytometry data illustrated the highest levels of CD11c+MHC‐II+ expression, and MIL‐88‐MG132@M treatment triggered abundant tumor‐associated antigenic stimulation of DCs and their presentation to lymphocytes, resulting in the proliferation and activation of antigen‐specific T‐ and B‐lymphocytes, initiating an adaptive immune response (Figure 8f). Thus, MIL‐88‐MG132@M treatment could transform tumor‐supportive chronic inflammation into immune‐activation‐associated acute inflammation, thereby suppressing tumor cell proliferation. Besides that, macrophage membrane coating prolonged the circulation time, improved drug retention levels, and maintained a long residence time in mCRC CT‐26 tumors [155].

Due to the COVID‐19 pandemic, the FDA granted many mRNA‐based vaccines emergency use authorization, and numerous mRNA therapies began to be explored for applications in cancer and cardiovascular diseases [156]. Cai et al. designed a novel mRNA delivery system using polylysine‐modified bovine serum albumin nanoparticles. Cationic protein nanoparticles (BSA‐PLL@MCM) were prepared and complexed with mRNA, followed by encapsulation with macrophage membranes to protect the mRNA from RNase degradation and enhance transfection efficiency in cancer cells. The transfection efficiency of BSA‐PLL@MCM@mRNA nanoparticles showed significant improvement in HepG2, MCF‐7, and RAW264.7 cells compared to immune cells [157].

Additionally, Liu et al. developed a neutrophil membrane‐coated ZIF‐8 nanoparticle delivery platform (AM@ZIF@NM) to target the delivery of anti‐microRNA‐155 (anti‐miR‐155) antisense oligonucleotides (ASOs) to endothelial cells in atherosclerotic lesions. The neutrophil membrane, which contains the membrane protein CD18, interacts with intercellular adhesion molecule‐1 (ICAM‐1) on the endothelial cell membrane, enhancing targeting efficiency. The delivery of anti‐miR‐155 effectively downregulated the expression of miR‐155 while rescuing the expression of its target gene BCL6. This anti‐miR‐155 nanoparticle therapy can suppress inflammatory responses in atherosclerotic lesions, alleviating the condition of atherosclerosis. This study demonstrates that immune cells, in addition to targeting and delivering therapeutics to cancer cells, can also address inflammatory responses in atherosclerotic lesions, offering a novel therapeutic approach for other diseases [70].

## 4 Stem Cell Membrane‐Camouflaged MOFs

**FIGURE 9:**
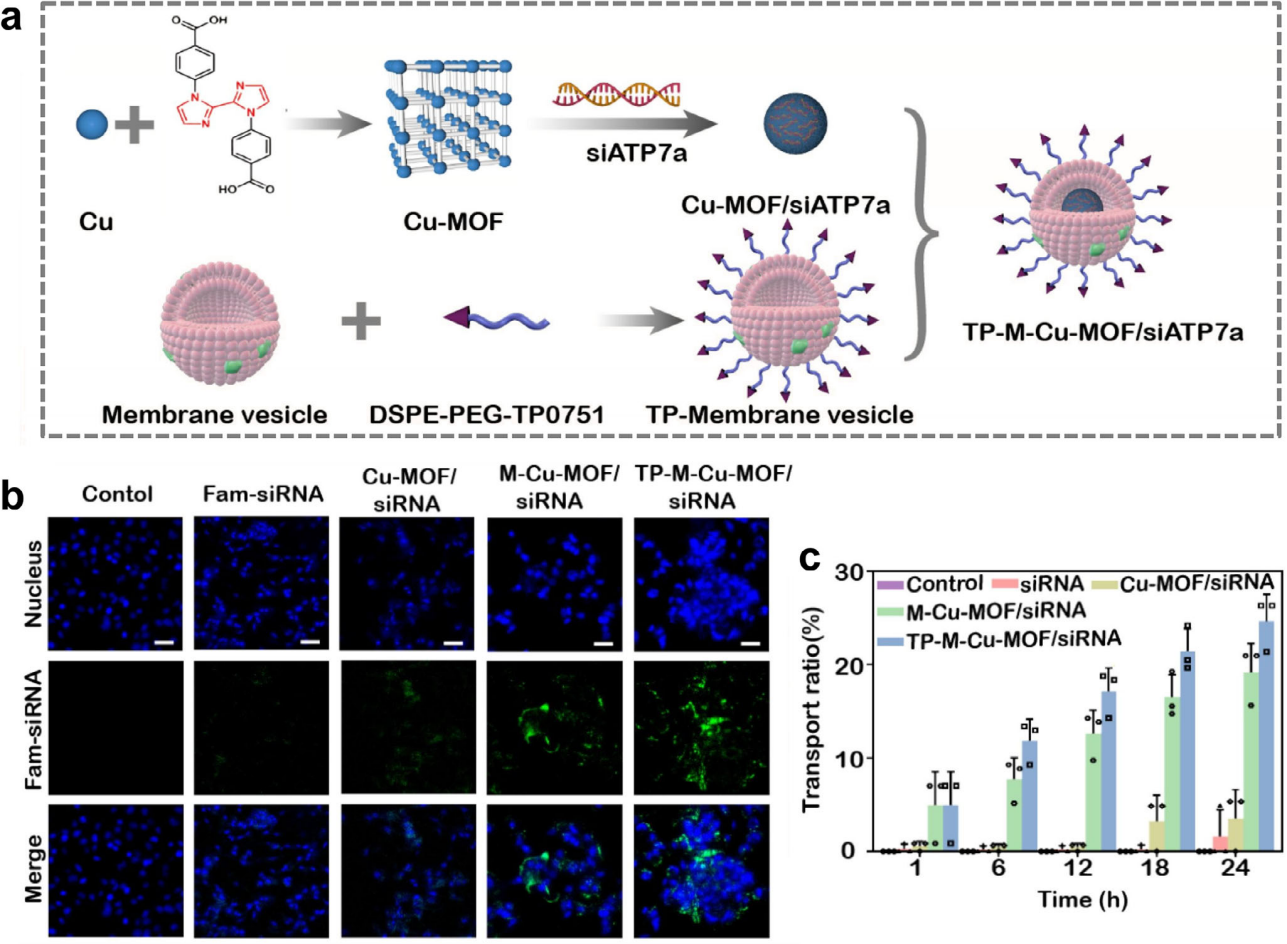
(a) Schematic illustration of the synthesis of TP‐M−Cu−MOF/siATP7a nanoparticles. (b) Fluorescent imaging of H69 cells treated with different nanoparticles. Scale bar: 30 µm. (c) Transport ratios of different nanoparticles across the in vitro BBB models [73]. Reproduced with permission. Copyright 2023, Elsevier.

Stem cells possess remarkable abilities of self‐renewal and multipotent differentiation, allowing them to migrate naturally to damaged tissues or lesions [158]. In the meantime, it has been determined that stem cell membranes with the addition of a syphilis mimetic peptide can confer nanoparticles with the capacity to effectively penetrate the blood‐brain barrier (BBB). Consequently, the combination of them with MOFs has been shown to be a highly effective strategy in the treatment of brain‐related tumors, including brain metastases. Zhang et al. constructed a Cu‐based MOF that was loaded with siRNA and camouflaged with mesenchymal stem cell membranes (MSC) that were decorated with the syphilis mimic TP0751 peptide in the outer layer (TP‐M‐Cu‐MOF/siATP7a) for brain metastases arising from small‐cell lung cancer (Figure 9a). TP‐M‐Cu‐MOF/siATP7a has been shown to accumulate and internalize at higher levels at the site of brain metastasis through BBB penetration and the biomimetic capacity of the MSC outer membrane (Figure 9b,c). Furthermore, Cu‐MOF has been shown to successfully protect siRNAs, which can activate gene silencing, copper efflux blockade, and lysine oxidase expression repression leading to cancer cell death, from lysosomal escape through the proton sponge effect in acidic conditions inside the tumors [73].

**FIGURE 10:**
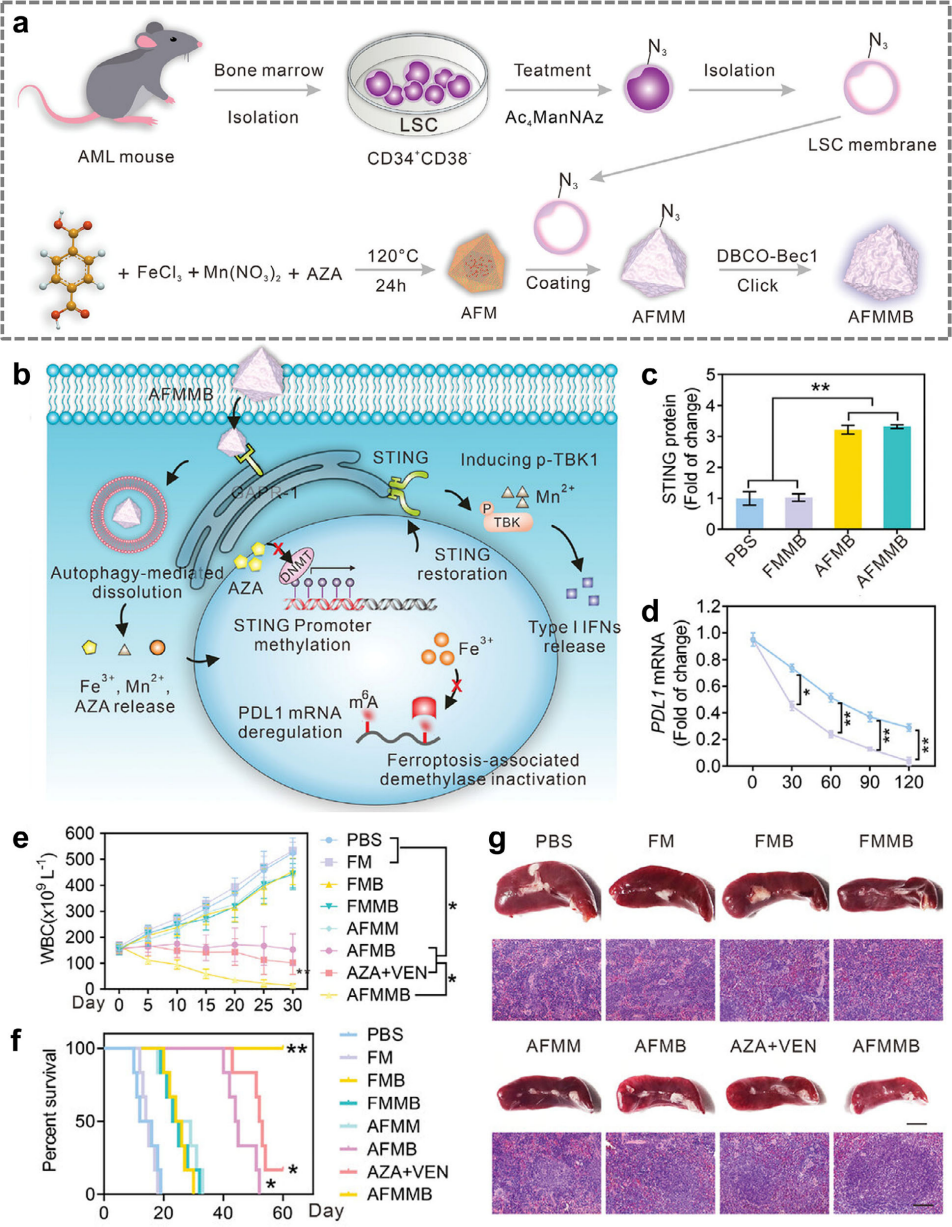
(a) Schematic diagram of the preparation of AFMMB. (b) Schematic diagram of intracellular molecular mechanism of AFMMB in vivo. (c) STING protein expression. (d) Effects of AFMMB on PD‐L1 mRNA stability. (e) WBC counts of leukemia‐bearing mice at different time points after different treatments. (f) Kaplan–Meier survival curves of mice receiving different treatments. (g) Images of spleen (Scale bar = 0.5 cm) and H&E‐stained spleen sections (Scale bar = 100 µm) after different treatments [75]. Reproduced with permission. Copyright 2023, Wiley.

Due to the extensive array of stem cells, a subset of these cells has been identified to possess distinctive targeting capabilities. For instance, dental pulp mesenchymal stem cell (DPSC) exhibits a propensity to be attracted to a chemokine known as CXCR8, which is secreted by oral squamous cell carcinoma (OSCC). Leveraging this observation, researchers have developed a novel approach involving the use of DPSC membrane‐modified MOF nanoparticles, which serve as a nanomedicine against tumor growth through the implementation of CDT [74]. Furthermore, leukemia stem cells (LSC) exhibit a robust homing tendency toward leukemia cells. Researchers integrated LSC membranes with bimetallic MOF (AFMMB), constructing a nanoparticle that yielded anti‐cancer effects by employing dual epigenetic alterations to enhance antitumor immunity from T‐cells (Figure 10a,b). The results demonstrated that AFMMB could effectively target AML and successfully regulate STING and PD‐L1 by modulating DNA and RNA methylation, thereby inhibiting cancer cell growth and prolonging the survival time of leukemia‐bearing mice effectively (Figure 10c–g) [75].

Stem cells have been shown to possess low immunogenicity when administered in vivo, a property that facilitates their evasion of rejection by the host immune system [159]. The encapsulation of nanoparticles within stem cells has been demonstrated to prolong the circulation time of the drug in the body, thereby preventing rapid clearance by the immune system. These distinctive characteristics position stem cells as a highly promising vehicle for drug delivery. Mu et al. utilized Fe_3_O_4_@PDA nanoparticles with excellent photothermal properties for efficient loading and delivery of siRNA, subsequently encapsulating these nanoparticles within MSC membranes [76]. The hydrophobic Fe_3_O_4_ nanoparticles, coated with PDA, exhibit remarkable near‐infrared (NIR) optical performance and high photothermal conversion efficiency, making them suitable photothermal therapy agents for in vivo cancer treatment [160]. Due to PDA's multiple surface functional groups, such as amine and catechol, it can effectively link with nucleic acids, facilitating siRNA delivery [161]. In their studies, Fe_3_O_4_@PDA‐siRNA@MSCs demonstrated significantly higher accumulation at tumor sites than Fe_3_O_4_@PDA alone, highlighting the extended circulation time and strong tumor‐targeting capability of MSCs. Under laser irradiation, DU145 cells cultured with Fe_3_O_4_@PDA‐siRNA@MSCs nanoparticles drastically decreased cell viability, dropping to 10%. In mouse cancer model experiments, treatment with Fe_3_O_4_@PDA‐siRNA@MSCs combined with laser exposure resulted in a 40% reduction in tumor volume [76]. Additionally, Mu et al. constructed a nanocarrier system comprising DOX and PD‐L1 siRNA encapsulated in MSC membranes (PDA‐DOX/siPD‐L1@SCM) for targeted treatment of prostate cancer bone metastasis [77]. Both in vitro and in vivo studies demonstrated the superior performance of PDA‐DOX/siPD‐L1@SCM nanoparticles in synergistic immunotherapy for prostate cancer bone metastasis [77]. Their work illustrates the potential of mesenchymal stem cell membranes as an alternative to polymer‐based encapsulation for siRNA, with promising applications in imaging‐guided photothermal therapy and gene therapy.

## 5 Bacterial Membrane‐Camouflaged MOFs

The role of microbes in the stimulation or inhibition of tumor growth is a subject of ongoing research. Historically, it has been found that some cancer patients with bacterial infections exhibited signs of tumor regression. This finding was further substantiated by the discovery of Coley's toxin, a bacterial extract capable of treating sarcomas, which demonstrated the capacity of specific microbes to combat cancer by activating anti‐tumor immune responses [162, 163, 164]. Specifically, it has been observed that Gram‐negative bacteria contain a multitude of pathogen‐associated molecular patterns (PAMPs), including peptidoglycan, lipopolysaccharide (LPS), and flagellin [165]. These PAMPs have the capacity to trigger an anti‐tumor immune response by binding to Toll‐like receptors (TLRs) and nucleotide‐binding oligomeric receptors (NLRs) on phagocytes [165, 166, 167]. However, the clinical application of live bacteria for the treatment of cancer remains problematic due to their uncontrollable proliferation [168, 169]. Fortunately, it has been demonstrated that the majority of bacteria possess the capacity to secrete membrane vesicles (MVs), which exhibit a robust innate immune signaling pathway activation capacity without the requirement for proliferation. A salient example of such MVs are the outer membrane vesicles (OMVs), which are spherical particles comprising lipopolysaccharide (LPS) and phospholipids. These are formed by the blebbing of the outer membrane of Gram‐negative bacteria [170, 171, 172, 173]. As a bacterial secretion pathway, studies have shown that MVs, exemplified by OMVs, are quantal delivery systems which can protect concentrated bioactive molecules from degradation for long traveling [174]. Consequently, researchers have exploited the properties of OMVs to integrate OMVs with multiple cancer therapies such as CAR‐T cells and nanomedicines, thereby constructing bacteria‐based immunotherapies and vaccines [175, 176, 177, 178, 179].

**FIGURE 11:**
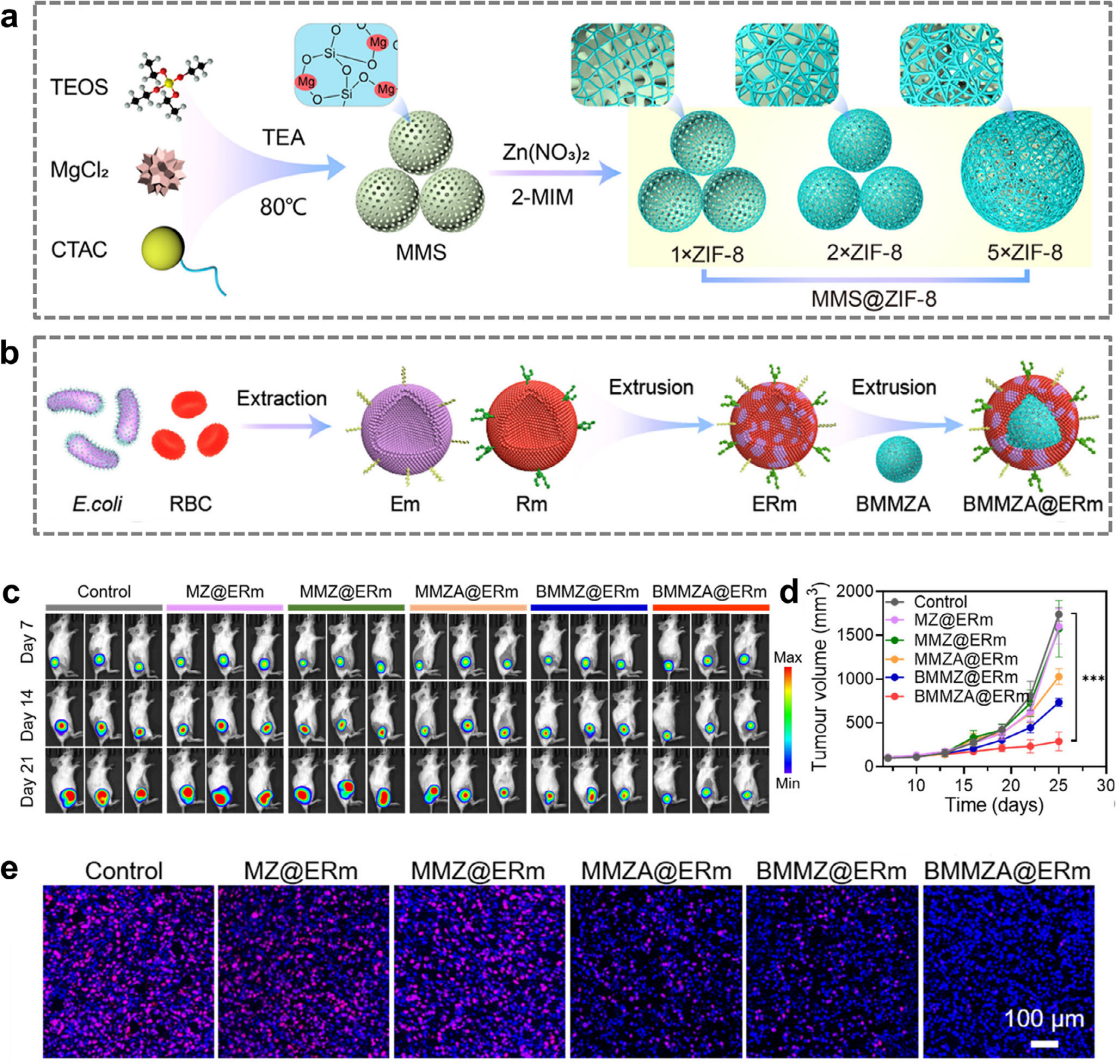
(a) Schematic diagram of the construction of MMS and MMS@ZIF‐8. (b) Schematic illustration of the synthesis of hybrid ERm and the construction of BMMZA@ERm. (c) In vivo bioluminescence imaging of 4T1‐luc tumor‐bearing mice after different treatments. (d) Tumor growth curves of 4T1‐luc tumor‐bearing mice. (e) Histological analyses with Ki67‐stained 4T1 tumors. Scale bar, 100 µm [78]. Reproduced with permission. Copyright 2024, American Chemical Society.

More recently, researchers have noted that encapsulating MOFs with MVs as camouflage not only improves drug delivery and targeting efficiency, but also that the combination of multiple therapeutic agents can achieve synergistic anti‐tumor effects. Yao et al. found that fusion of *E. coli* OMVs with RBC membranes (ERm) could effectively improve the stability of OMVs in bloodstream without weakening its anti‐tumor capacity, so they first loaded magnesium‐doped mesoporous silica particles (MMS) with DOX and bortezomib (BTZ), and then decorated the silica particles with ZIF‐8 containing ascorbic acid (AA) to form BMM@ZIF‐AA (BMMZA), followed by encapsulating BMMZA with the hybrid membranes of OMVs and RBC (BMMZA@ERm, Figure 11a,b) to construct a nano‐turret for chemotherapy‐assisted ascorbate‐mediated immunotherapy (CAMIT). As demonstrated in Figure 11c, the existence of OMVs and RBC hybrid membranes enhanced the targeting efficiency of nanoparticles to tumor while minimizing the impact on liver. More importantly, BMZZA@ERm showed the strongest tumor inhibition rate and efficiency compared to all controls with separate missing components (Figure 11d,e). This phenomenon demonstrated that the presence of either drug molecules or hybrid membranes has a significant impact on the synergistic therapeutic efficacy of CAMIT. Specifically, the presence of pH‐sensitive ZIF‐8 ensures that AA was released only in the acidic tumor microenvironment, while the coexistence of OMV with Mg^2+^ continuously released from MMS further enhanced the AA‐associated immunotherapy [78].

**FIGURE 12:**
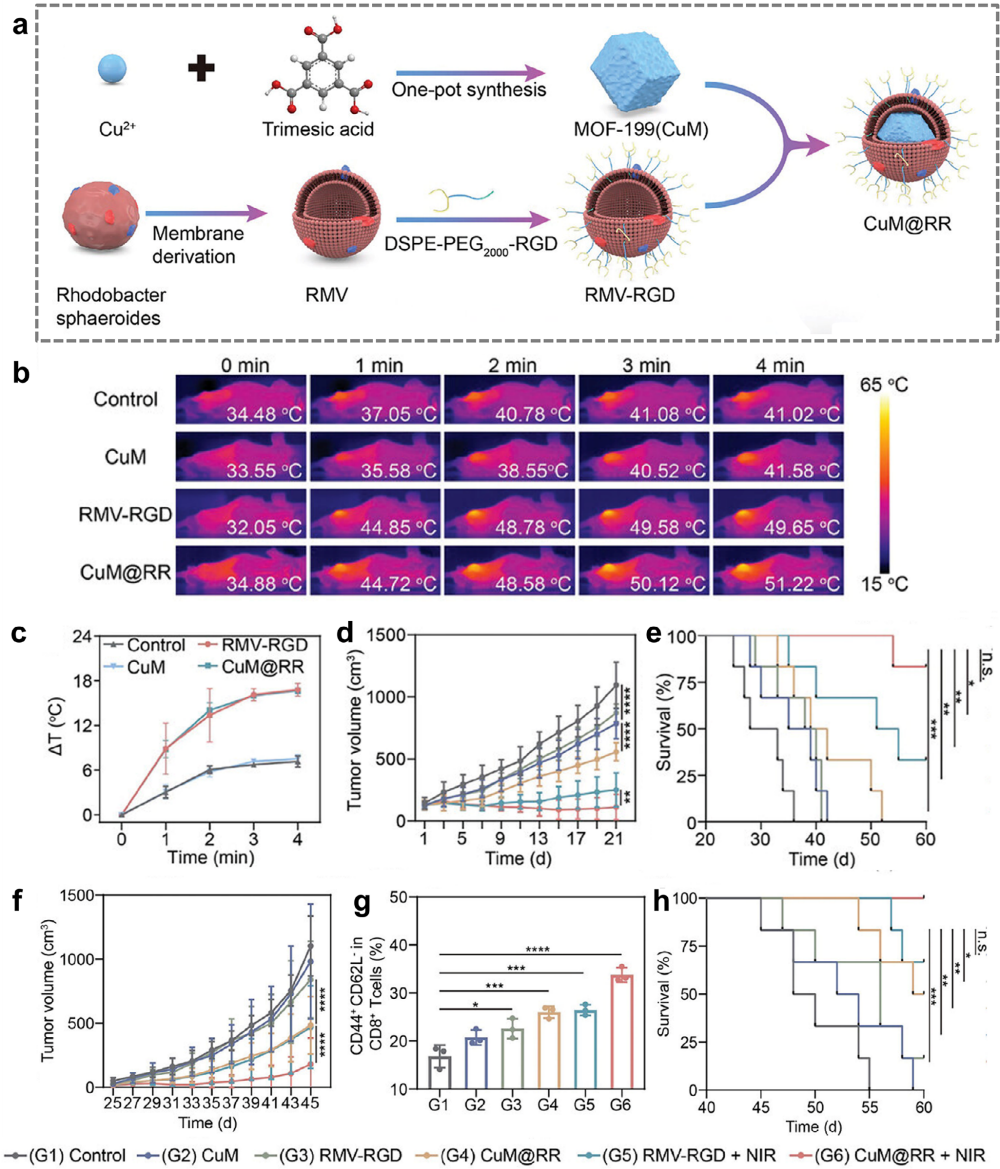
(a) Schematic illustration of the construction of CuM@RR. (b) Representative infrared thermal images of murine 4T1 breast tumor‐bearing mice after treating with CuM, RMV‐RGD or CuM@RR. (c) Quantitative analysis of the change of temperature. (d) Tumor growth curves and (e) survival rate of mice after different treatments. (f) Growth curves of rechallenge tumors. (g) Quantitative analysis of T_EM_ cells in the spleens. (h) Survival curves for tumor‐rechallenged mice after different treatments [79]. Reproduced with permission. Copyright 2025, Wiley.

Besides, the immunotherapeutic effect of MVs‐camouflaged MOFs nanocomposites can be further enhanced by taking advantage of favorable properties of bacteria other than their anti‐tumor functions. For example, *Rhodobacter sphaeroides* is a photosynthetic bacterium which can produce endogenous bacteriochlorophyll a (Bchl a). The existence of Bchl a confers upon the bacterium a distinct characteristic absorption peak in near infrared (NIR) wavelength range of 800–860 nm [180]. A substantial body of research has previously been conducted on *Rhodobacter sphaeroides* in the context of PTT [181, 182]. In a recent study, Niu et al. investigated the MVs of this bacterium (RMVs) in depth and discovered that the RMVs also contain Bchl a and exhibit analogous optical characteristics in the NIR region as the bacterium itself [79, 183, 184]. Hence, they developed a multifunctional nano‐biohybrid (CuM@RR), as shown in Figure 12a, which combines Cu‐MOF with Cu^2+^ releasing ability and DSPE‐PEG2000‐RGD functionalized RMVs with NIR optical properties resulted in a multifunctional and effective anti‐malignant solid tumors nanocomposite for cuproptosis, PTT and immunotherapy. The results showed that CuM@RR manifested the most pronounced PTT effect when exposed to 808 nm NIR laser irradiation, with the temperature of the tumor tissue rising to over 50°C within approximately 4 min (Figure 12b,c), and cytokine levels of IL‐6, IL‐12p70, TNF‐𝛼, and INF‐𝛾 were substantially elevated in the tumor tissue. Concurrently, the tumor growth rate in murine 4T1 breast tumor‐bearing mice was significantly reduced in short‐term, long‐term or rechallenge‐tumor evaluations when compared to other control subjects (Figure 12d,f) as well as the immune memory response has also been enhanced (Figure 12g). The survival rate of mice has greatly improved, and the biosafety of nanomaterials has also been confirmed in both primary and recurrent tumors (Figure 12e,h) [79]. Research on bacteria‐based immunotherapy has been ongoing for a considerable period. However, recent investigations into the use of bacterial MVs in conjunction with MOF for anti‐tumor purposes are in their nascent stages. A limited number of findings have already demonstrated the efficacy of such nanocomposites in cancer therapy, suggesting a promising future for further research in this area.

## 6 Challenges and Perspectives

While the short‐term biocompatibility of cell membrane‐coated MOFs has been widely demonstrated, the long‐term fate of their degradation products remains a critical knowledge gap. Upon intracellular uptake and lysosomal escape, the MOF core disassembles into constituent metal ions (Zn^2^^+^, Zr^4^^+^) and organic linkers (e.g., 2‐methylimidazole). Although ions like zinc are essential trace elements, their local accumulation at high concentrations could induce oxidative stress or interfere with enzymatic homeostasis in metabolic organs such as liver and kidneys. Furthermore, the clearance kinetics of organic linkers are often non‐linear, if not efficiently renally excreted, they may pose risks of chronic nephrotoxicity. Thus, future studies must transition from acute survival assays to long‐term pharmacokinetics studies using radiolabeling techniques to track the biodistribution and excretion rates of both the metal nodes and the organic ligands independently.

A paramount concern often overshadowed by the success of “immune evasion” is the potential immunogenicity of the membrane proteins. The top‐down extraction process may alter the quaternary structure of membrane proteins, exposing cryptic epitopes that the immune system recognizes as “non‐self”, potentially triggering anaphylaxis upon repeated administration. Specifically for cancer cell membranes (CCMs) or allogeneic membranes, there is a theoretical risk that introducing these exogenous antigens could trigger autoimmune responses against healthy tissues sharing similar epitopes or induce a “cytokine storm” in sensitive patients. Looking forward, the inherent intra‐tumor and inter‐tumor heterogeneity remain formidable barriers for single‐membrane coating strategies [185]. A “one‐size‐fits‐all” membrane often fails to address the divergent molecular profiles of metastatic sub‐clones, potentially leading to immune escape and therapeutic resistance. Future research should pivot toward hybrid membrane systems—fusing disparate cellular functionalities (e.g., combining the immune‐evasive properties of RBCs with the homotypic targeting of CCMs) or employing genetically engineered cell lines to display multi‐valent ligands [186, 187]. Such synergistic biomimetic coatings, integrated with the stimuli‐responsive nature of MOF cores, will be essential for achieving comprehensive eradication of heterogeneous and metastatic malignancies. Furthermore, metabolic glycoengineering can be implemented to modify functional groups on the membrane surface, thereby endowing biomimetic MOFs with more proactive and precise molecular recognition capabilities. A comprehensive comparison of mainstream cell membrane coatings is presented in Table 2. The summarized trade‐offs between their targeting efficiency and safety profiles underscore the growing necessity for developing hybrid or genetically engineered membrane systems.

**TABLE 2:** The summary of cell membrane coating types.

Membrane Source	Key Advantages	Limitations & Challenges	Application	Refs
Red Blood Cell Membrane	Long Circulation, membrane‐expressed CD47 contributes to immune evasion.	Lack of tumor targeting, may induce hemolytic reactions	Prolonging the half‐life of chemotherapeutics	[188]
Cancer Cell Membrane	Homologous targeting, stimulate tumor‐specific immunity	Shorter circulation, risk of transferring genetic material or malignant properties	Tumor‐specific imaging and diagnostics	[189]
Immune Cell Membrane	Barrier penetration, Inflammation targeting, Immune evasion	Extraction difficulty, may induce unwanted immune activation or inflammation	Targeting circulating tumor cells and solid tumors	[190, 191]
Stem Cell Membrane	Tumor Homing, low immunogenicity and anti‐inflammatory properties	Low specificity, difficult to scale up, limited drug‐loading capacity	Neural stem cells can cross the BBB via "stem cell homing"	[192, 193]
Bacterial Membrane	Strong stimulation of innate and adaptive immune systems	High risk of toxicity, requires careful removal of peptidoglycans and toxins	Active targeting of bacterial infections	[194, 195]

The current source of cell membranes and the stability of membrane proteins are issues that can lead to poor reproducibility of such nanoplatform. It is also worthwhile to consider the formation mechanism of the cell membrane coating MOFs and whether different interactions between membranes and MOFs, such as physical adsorption, hydrophobic interactions, or electrostatic interactions, have an impact on the stability and integrity of the cell membrane coatings. In the context of dynamic stability, cell membranes can be rendered more stable through surface modifications, thereby ensuring their integrity during cellular uptake and long‐term internal cycling. Moreover, the safety of the source of cell membranes must also be emphasized, and it is worth noting whether exogenous cell membranes pose risks such as infectious diseases.

**FIGURE 13:**
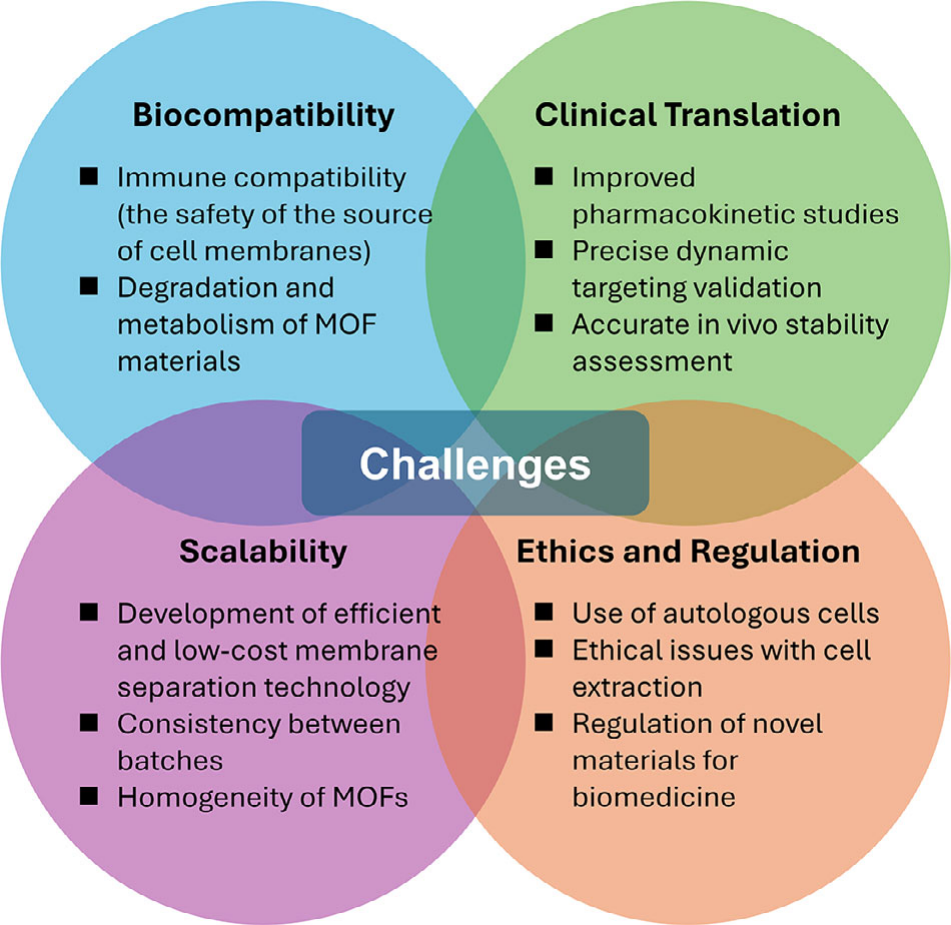
Challenges in the future development of biomimetic MOFs in cancer therapy.

Furthermore, the stability and structural integrity of the biomimetic interface present significant challenges. Conventional preparation methods, such as physical extrusion or sonication, frequently lead to membrane protein denaturation or patchy coating, which predisposes the nanoplatform to membrane detachment during complex systemic circulation. To overcome these hurdles, future research should pivot toward advanced manufacturing processes and interfacial chemical modulation. For instance, microfluidics can be employed to achieve precise layer‐by‐layer assembly of core‐shell structures, ensuring coating homogeneity. Alternatively, the incorporation of chemical crosslinkers or cholesterol doping strategies can be utilized to enhance membrane rigidity and shear resistance. The challenges in the development of biomimetic MOFs in the field of cancer therapy are summarized in Figure 13.

## 7 Conclusion

The emergence of biomimetic MOFs provides a brand‐new idea for the development of cancer therapeutics, which combines the flexible, versatile, stable, and structurally unique features of MOFs with the targeting and camouflage capabilities of liposomes or cell membranes to maximize the effectiveness of cancer therapy. At the same time, because of the flexibility of the composition of the MOFs, this type of drug delivery system can combine multiple therapeutic approaches, eliminating the drawbacks of the low efficiency of single cancer therapy and killing cancer cells in an all‐round way through multiple therapeutic modalities with minimal side effects.

In conclusion, MOFs with biomimetic strategies have a promising future in cancer therapy, while researchers should maintain a rigorous and cautious attitude and pay attention to the problems these complex biocomposites may bring to ensure that biomimetic MOFs play their proper roles in disease treatment areas. Future research should not be limited to the mere superposition of materials but should instead strive to decipher the intricate in vivo biophysical behaviors of this interface. Only by deeply integrating immunological characteristics, metabolic kinetics, and material engineering at the inception of design—while maintaining a rigorous approach to addressing biocompatibility and manufacturing controllability—can biomimetic MOFs truly realize their immense potential in precision oncology.

## Conflicts of Interest

The authors declare no conflict of interest.
